# The global burden of disease attributable to high body mass index in 195 countries and territories, 1990–2017: An analysis of the Global Burden of Disease Study

**DOI:** 10.1371/journal.pmed.1003198

**Published:** 2020-07-28

**Authors:** Haijiang Dai, Tariq A. Alsalhe, Nasr Chalghaf, Matteo Riccò, Nicola Luigi Bragazzi, Jianhong Wu

**Affiliations:** 1 Department of Cardiology, Third Xiangya Hospital, Central South University, Changsha, China; 2 Laboratory for Industrial and Applied Mathematics, Centre for Disease Modelling, Department of Mathematics and Statistics, York University, Toronto, Ontario, Canada; 3 College of Sport Sciences and Physical Activity, King Saud University, Riyadh, Saudi Arabia; 4 Group for the Study of Development and Social Environment, Faculty of Letters and Human Sciences of Sfax, University of Sfax, Sfax, Tunisia; 5 Higher Institute of Sport and Physical Education of Sfax, University of Sfax, Sfax, Tunisia; 6 Occupational Health and Safety Services, Department of Public Health, AUSL-IRCCS di Reggio Emilia, Reggio Emilia, Italy; University of Cambridge, UNITED KINGDOM

## Abstract

**Background:**

Obesity represents an urgent problem that needs to be properly addressed, especially among children. Public and global health policy- and decision-makers need timely, reliable quantitative information to develop effective interventions aimed at counteracting the burden generated by high body mass index (BMI). Few studies have assessed the high-BMI-related burden on a global scale.

**Methods and findings:**

Following the methodology framework and analytical strategies used in the Global Burden of Disease Study (GBD) 2017, the global deaths and disability-adjusted life years (DALYs) attributable to high BMI were analyzed by age, sex, year, and geographical location and by Socio-demographic Index (SDI). All causes of death and DALYs estimated in GBD 2017 were organized into 4 hierarchical levels: level 1 contained 3 broad cause groupings, level 2 included more specific categories within the level 1 groupings, level 3 comprised more detailed causes within the level 2 categories, and level 4 included sub-causes of some level 3 causes. From 1990 to 2017, the global deaths and DALYs attributable to high BMI have more than doubled for both females and males. However, during the study period, the age-standardized rate of high-BMI-related deaths remained stable for females and only increased by 14.5% for males, and the age-standardized rate of high-BMI-related DALYs only increased by 12.7% for females and 26.8% for males. In 2017, the 6 leading GBD level 3 causes of high-BMI-related DALYs were ischemic heart disease, stroke, diabetes mellitus, chronic kidney disease, hypertensive heart disease, and low back pain. For most GBD level 3 causes of high-BMI-related DALYs, high-income North America had the highest attributable proportions of age-standardized DALYs due to high BMI among the 21 GBD regions in both sexes, whereas the lowest attributable proportions were observed in high-income Asia Pacific for females and in eastern sub-Saharan Africa for males. The association between SDI and high-BMI-related DALYs suggested that the lowest age-standardized DALY rates were found in countries in the low-SDI quintile and high-SDI quintile in 2017, and from 1990 to 2017, the age-standardized DALY rates tended to increase in regions with the lowest SDI, but declined in regions with the highest SDI, with the exception of high-income North America. The study’s main limitations included the use of information collected from some self-reported data, the employment of cutoff values that may not be adequate for all populations and groups at risk, and the use of a metric that cannot distinguish between lean and fat mass.

**Conclusions:**

In this study, we observed that the number of global deaths and DALYs attributable to high BMI has substantially increased between 1990 and 2017. Successful population-wide initiatives targeting high BMI may mitigate the burden of a wide range of diseases. Given the large variations in high-BMI-related burden of disease by SDI, future strategies to prevent and reduce the burden should be developed and implemented based on country-specific development status.

## Abstract 2

Nicola Bragazzi and colleagues estimate the global burden of high BMI related diseases using data from the Global Burden of Disease study 2017

## Author summary

**Why was this study done?:**

- Obesity represents an urgent issue that needs to be properly addressed. A successful response to the challenge of obesity requires an accurate understanding of its current burden.
- Few studies have assessed the disease burden attributable to high body mass index (BMI) at the global level.
- To better inform prevention and intervention strategies, we aimed to provide comprehensive and updated information about the global disease burden attributable to high BMI in 195 countries and territories.

**What did the researchers do and find?:**

- We used the modeling framework in the Global Burden of Disease Study 2017 to examine the global deaths and disability-adjusted life years (DALYs) attributable to high BMI.
- Globally, in 2017, high BMI caused 2.4 million (95% UI 1.6 million, 3.4 million) deaths and 70.7 million (95% UI 49.1 million, 94.9 million) DALYs in females, and 2.3 million (95% UI 1.4 million, 3.4 million) deaths and 77.0 million (95% UI 49.7 million, 108.2 million) DALYs in males.
- Between 1990 and 2017, despite the fact that the age-standardized rate of high-BMI-related DALYs increased by only 12.7% for females and 26.8% for males, the global number of high-BMI-related DALYs has more than doubled for both sexes.
- Cardiovascular disease was the leading cause of high-BMI-related DALYs, followed by diabetes and kidney diseases, and neoplasms; they together accounted for 89.3% of all high-BMI-related DALYs.
- The disease burden attributable to high BMI and its temporal changes varied widely by Socio-demographic Index.

**What do these findings mean?:**

- Our results suggest that high BMI remains a major global health challenge. Successful population-wide initiatives targeting high BMI may mitigate the burden of a wide range of diseases, such as cardiovascular disease, diabetes and kidney diseases, and neoplasms.
- Policy-makers should take into account country-specific development status when developing and implementing strategies to prevent and reduce the disease burden generated by high BMI.

## Introduction

The interplay of genetic and behavioral factors, such as unhealthy lifestyle habits, can lead to an increase in non-communicable disorders, including cancer, type 2 diabetes, hypertension, dyslipidemia, and osteoarthritis, as well as other chronic or degenerative diseases [1–3]. Among these, obesity is particularly widespread on the global scale, resulting in an epidemic. According to some estimates, the annual burden of disease imposed by high body mass index (BMI) can be quantified as an estimated 216,000 deaths and US$113.9 billion in direct healthcare costs in the US alone [4,5].

Obesity represents an urgent issue that needs to be properly addressed, especially among children [6]. However, despite the importance of this issue, behavioral risk factors associated with obesity rarely receive the attention they deserve: Insufficient focus during medical training is given to these factors, whereas great emphasis is given to the biological aspects of diseases, overlooking the psychological, behavioral, and social determinants of health [7]. The same holds among public and global health policy- and decision-makers, who decide to allocate few financial resources to the field of behavioral medicine, prioritizing, instead, other clinical fields and disciplines [8].

A successful response to the challenge of obesity needs timely, reliable quantitative information to design ad hoc, effective interventions aimed at counteracting the disease burden generated by high BMI. These interventions can be combined with pharmacotherapy and can be complex, multi-domain programs targeting different, sometimes interrelated and overlapping risk factors [9]. Few studies so far have assessed the global changing patterns of obesity. The collaborative groups of the Global Burden of Disease Study (GBD) [10] have collected data from 68.5 million children and adults between 1980 and 2015 from over 195 countries and territories. Authors have found that the prevalence rate of obesity has rapidly doubled in more than 70 countries and has continuously increased in most other countries, causing 4.0 million deaths, two-thirds of which were due to cardiovascular disease.

Due to the dearth of currently available data, especially on the global scale, the present study, adopting the methodology framework and analytical strategies of the GBD collaborative groups, aimed to update the previous GBD investigation, in order to provide stakeholders with updated information to better inform their decisions and the policies to implement.

## Methods

### Data sources

Data on the burden of disease attributable to high BMI were obtained from the Global Health Data Exchange GBD Results Tool (http://ghdx.healthdata.org/gbd-results-tool), which was created by GBD collaborators to provide a systematic assessment of age- and sex-specific mortality for 282 causes, prevalence and years lived with disability for 354 diseases and injuries, and comparative risks for 84 risk factors in 195 countries and territories, from 1 January 1990 to 31 December 2017. Detailed methodologies of GBD 2017 and the comparative risk assessment specifically for high BMI have been described elsewhere [11–13]. The protocol used for GBD 2017 was posted on the website of the Institute for Health Metrics and Evaluation [14]. Because GBD 2017 uses de-identified, aggregated data, a waiver of informed consent was reviewed and approved by the University of Washington Institutional Review Board [15]. This study is compliant with the Guidelines for Accurate and Transparent Health Estimates Reporting (S1 GATHER Checklist).

### Definitions

High BMI was defined as BMI ≥ 25 kg/m^2^ for adults (aged 20+ years) and using thresholds from the International Obesity Task Force standards for children (aged <20 years) [11]. GBD 2017 incorporated data on high BMI from 2,288 data sources (http://ghdx.healthdata.org/gbd-2017/data-input-sources). Detailed information about the process of data selection and data inputs has been published previously [11].

Cause-specific deaths and disability-adjusted life years (DALYs) by age, sex, year, and location were collected from GBD 2017. The DALY is a summary measure that quantifies the overall burden of disease [11–13]. It represents the sum of years of life lost due to premature death and years lived with disability. With the DALY, the burden of disease that causes premature death but little disability can be compared to burden of disease that only causes disability but no death. One DALY can be regarded as the loss of 1 year in full health [16]. GBD 2017 modeling strategies for estimating cause-specific deaths and DALYs have been described in detail elsewhere [12,13].

In GBD 2017, causes of death and DALYs were classified into 4 levels, which are mutually exclusive and collectively exhaustive [12,17]. The 3 cause groupings at level 1 were communicable, maternal, and neonatal conditions and nutritional disease; non-communicable diseases; and injuries. At level 2, there were 22 disease and injury categories within the level 1 groupings, such as cardiovascular disease within non-communicable diseases. Level 3 represented more detailed causes within the level 2 categories, such as stroke within cardiovascular disease. Level 4 included sub-causes of some level 3 causes, such as ischemic stroke within stroke. In GBD 2017, high BMI proved to be associated with 6 level 2 causes of death and 8 level 2 causes of DALYs in both sexes. Moreover, the number of causes of death attributable to high BMI was 22 at level 3 and 34 at level 4 in females, and 19 at level 3 and 31 at level 4 in males. The number of causes of DALYs attributable to high BMI was 26 at level 3 and 38 at level 4 in females, and 23 at level 3 and 35 at level 4 in males.

Socio-demographic Index (SDI), a composite indicator of a geographical location’s development status, was calculated based on total fertility rate among females younger than 25 years, educational attainment for those aged 15 years or older, and lag distributed income per capita [11–13]. SDI ranged from 0 to 1, where 0 represents the minimum level of development, and 1 represents the maximum level of development. The 195 countries and territories were categorized according to SDI quintile into 5 groups: low-SDI, low-middle-SDI, middle-SDI, high-middle-SDI, and high-SDI quintile.

### Statistical analysis

We computed the number of deaths or DALYs, age-standardized rate, and percent change with 95% uncertainty intervals (UIs) to quantify the burden of disease attributable to high BMI, overall and by age, sex, year, and location. Age-standardized rate was calculated by standardization to the global age structure, and this standardization was considered to be necessary when comparing populations from different locations or a sample population over time [18]. UIs were calculated from 1,000 draw-level estimates for each parameter, and 95% UIs were defined by the 25th and 975th values of the ordered 1,000 estimates. The attributable proportions of age-standardized DALYs due to high BMI were measured using population attributable fractions, which represent the age-standardized DALYs that could have been avoided if the exposure to high BMI was reduced to an alternative ideal exposure scenario. Population attributable fractions were estimated using the GBD 2017 comparative risk assessment approach that has been described previously [11]. Finally, we examined the relationship between SDI and the burden of disease attributable to high BMI, by location and year. The detailed analytical methods used for this study have been published previously [11–13], and the related codes can be accessed at http://ghdx.healthdata.org/gbd-2017/code. A 95% UI excluding 0 was considered to be statistically significant.

## Results

### Overall impact of high BMI

Between 1990 and 2017, the global deaths and DALYs attributable to high BMI have more than doubled for both females and males (S1 Fig). The global deaths attributable to high BMI have increased from 1.2 million (95% UI 0.7 million, 1.8 million) in 1990 to 2.4 million (95% UI 1.6 million, 3.4 million) in 2017 for females, and have increased from 1.0 million (95% UI 0.5 million, 1.6 million) in 1990 to 2.3 million (95% UI 1.4 million, 3.4 million) in 2017 for males. The global DALYs attributable to high BMI have increased from 33.1 million (95% UI 20.0 million, 48.8 million) in 1990 to 70.7 million (95% UI 49.1 million, 94.9 million) in 2017 for females, and have increased from 31.9 million (95% UI 16.9 million, 51.2 million) in 1990 to 77.0 million (95% UI 49.7 million, 108.2 million) in 2017 for males. Nevertheless, between 1990 and 2017, the age-standardized rate of high-BMI-related deaths remained stable for females and only increased by 14.5% for males, and the age-standardized rate of high-BMI-related DALYs only increased by 12.7% for females and 26.8% for males (S2 Fig).

**Fig 1:**
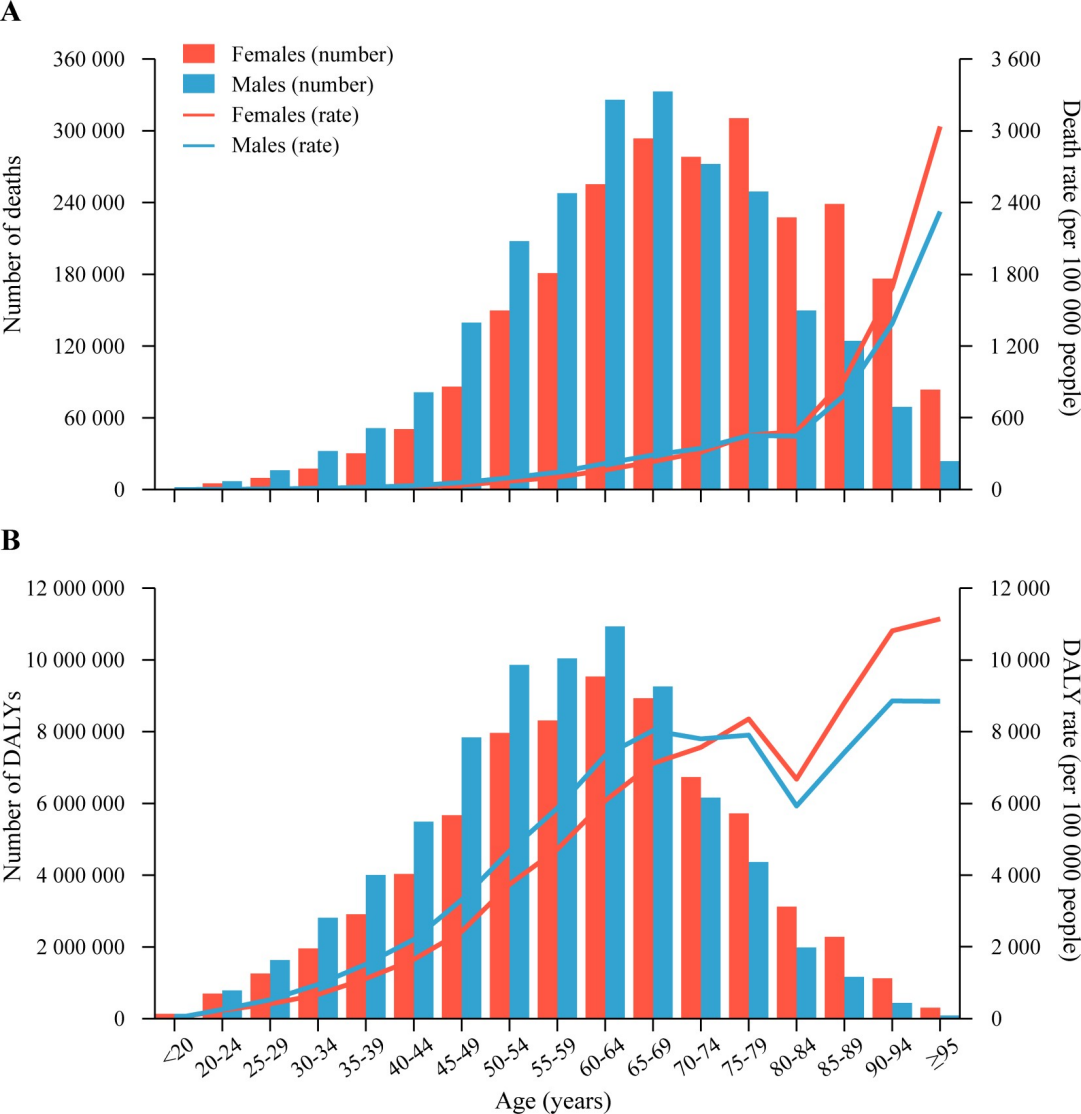
Age-specific numbers and rates of deaths and DALYs attributable to high body mass index by sex, in 2017. (A) Deaths. (B) DALYs. DALY, disability-adjusted life year.

Age-specific rates of high-BMI-related deaths and DALYs increased with increasing age, with the exception of DALYs in people aged 75–84 years; this pattern was similar for females and males (Fig 1). Both high-BMI-related death and DALY rates were lower in females in age groups younger than 75 years than in males in the same age groups, whereas the rates were higher in females than in males in age groups ≥75 years. The number of high-BMI-related deaths peaked in the age group 75–79 years in females, whereas the peak in males was observed in the age group 65–69 years. The number of high-BMI-related DALYs peaked in the age group 60–64 years in both sexes. Furthermore, the numbers of high-BMI-related deaths and DALYs were both lower in females than in males in age groups <70 years, whereas the numbers were higher in females than in males in age groups ≥70 years.

Across the 21 GBD regions, in 2017, the highest age-standardized rates of high-BMI-related deaths and DALYs were observed in Oceania (171.7 [95% UI 104.0, 251.2] deaths per 100,000 people and 5,779.9 [95% UI 3,671.5, 8,102.7] DALYs per 100,000 people; S1 Table). The lowest rates of high-BMI-related deaths and DALYs were observed in high-income Asia Pacific (15.4 [95% UI 6.2, 27.0] deaths per 100,000 people and 576.0 [95% UI 253.9, 973.1] DALYs per 100,000 people). From 1990 to 2017, the region with the largest percentage increase in the rates of high-BMI-related deaths and DALYs was South Asia (158.3% [95% UI 77.3%, 403.9%] for deaths and 165.9% [95% UI 83.3%, 400.7%] for DALYs), whereas the region with the biggest percentage decline was high-income Asia Pacific (−39.2% [95% UI −46.3%, −22.9%] for deaths and −23.9% [95% UI (−32.7%, −3.1%] for DALYs; S1 Table).

**Fig 2:**
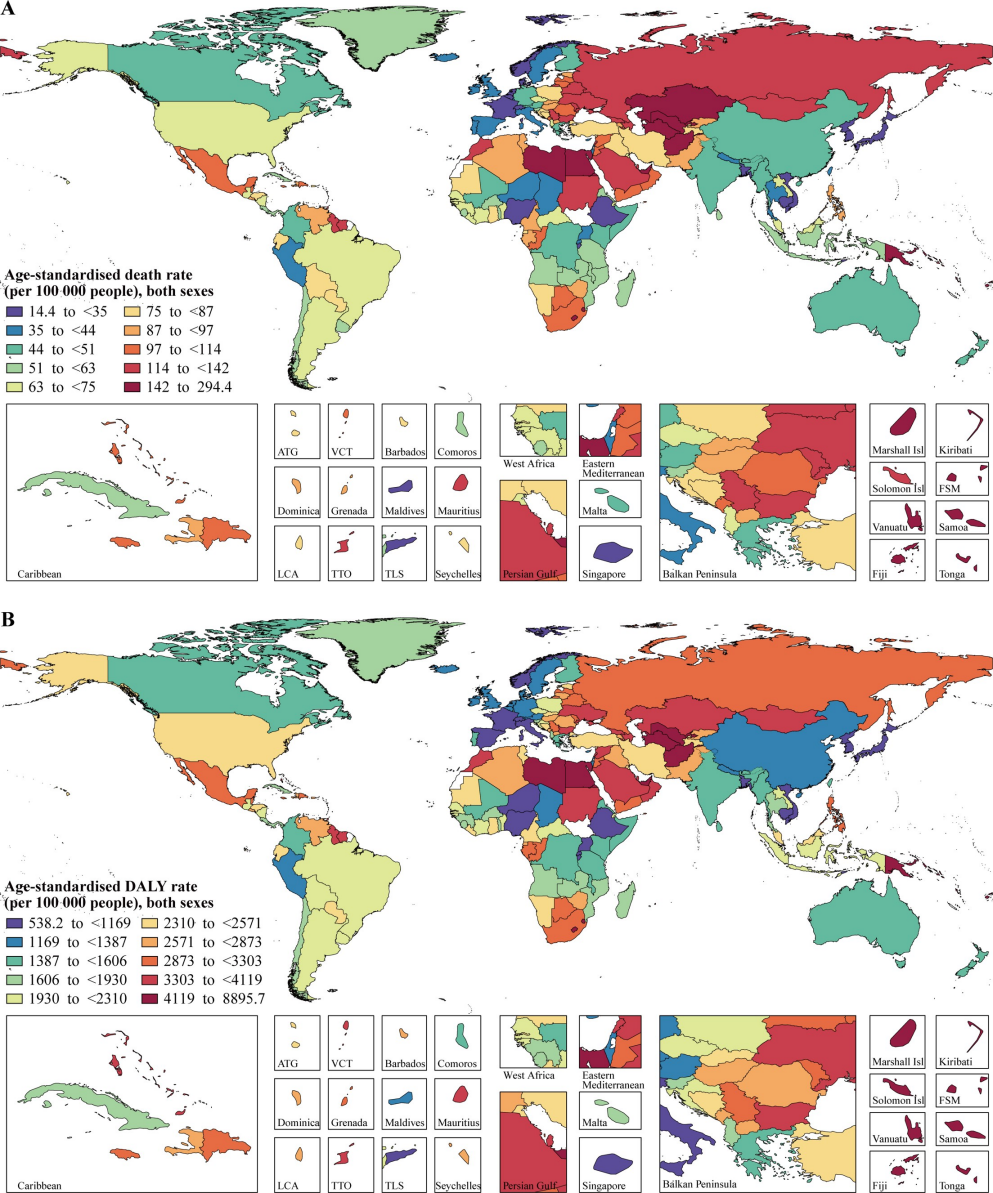
Age-standardized death and DALY rates attributable to high body mass index for both sexes combined in 2017. (A) Deaths. (B) DALYs. The maps were drawn by the R package “maptools” with the shapefiles edited manually in ArcMap. ATG, Antigua and Barbuda; Isl, Islands; FSM, Federated States of Micronesia; LCA, Saint Lucia; TLS, Timor-Leste; TTO, Trinidad and Tobago; VCT, Saint Vincent and the Grenadines; DALY, disability-adjusted life year.

In 2017, the country with the highest age-standardized rates of high-BMI-related deaths and DALYs was Fiji (294.4 [95% UI 209.5, 379.8] deaths per 100,000 people and 8,895.7 [95% UI 6,817.2, 11,004.9] DALYs per 100,000 people), whereas the country with the lowest rates was Japan (14.4 [95% UI 5.9, 25.6] deaths per 100,000 people and 538.2 [95% UI 235.9, 915.6] DALYs per 100,000 people; Fig 2; S1 Table). Among the world’s 20 most populous countries, Egypt had the highest age-standardized rates of high-BMI-related deaths (187.3 [95% UI 120.0, 256.6] per 100,000 people) and DALYs (5,322.5 [95% UI 3,600.1, 7,037.4] per 100,000 people), and Japan had the lowest rates of high-BMI-related deaths and DALYs. From 1990 to 2017, Bangladesh, one of the world’s 20 most populous countries, experienced the largest percentage increase in the rates of high-BMI-related deaths (190.3% [95% UI 73.7%, 774.0%]) and DALYs (231.8% [95% UI 97.2%, 915.8%]) among all countries, whereas Iraq experienced the biggest percentage decline among all countries (−60.0% [95% UI −65.3%, −53.3%] for deaths and −53.0% [95% UI −59.4%, −45.6%] for DALYs). Turkey was the country with the biggest percentage decline in the rates of high-BMI-related deaths (−35.0% [95% UI −43.1%, −24.6%]) and DALYs (−28.4% [95% UI −36.9%, −17.3%]) among the world’s 20 most populous countries.

### Impact of high BMI on each disease

In 2017, cardiovascular disease was the leading cause of high-BMI-related DALYs (992.4 [95% UI 649.7, 1,363.9] per 100,000 people; Table 1), followed by diabetes and kidney diseases (496.7 [95% UI 347.2, 679.6] per 100,000 people) and neoplasms (133.4 [95% UI 76.5, 205.7] per 100,000 people); they together accounted for 89.3% of all high-BMI-related DALYs. Similar patterns were observed for deaths. Other GBD level 2 causes of high-BMI-related DALYs and deaths are shown in Table 1, S1 Fig, and S2 Fig. Additionally, of all cardiovascular disease age-standardized DALYs worldwide, 21.6% (95% UI 14.0%, 29.7%) was attributable to high BMI; the corresponding proportions were 38.3% (95% UI 28.4%, 48.7%) for diabetes and kidney diseases and 4.6% (95% UI 2.7%, 7.1%) for neoplasms.

**Table 1:** Global deaths and DALYs attributable to high body mass index for both sexes combined in 2017 and percentage change from 1990 to 2017.

Cause of death or DALYs	Deaths				DALYs			
	2017 age-standardized rate per 100,000 people	Percentage change in age-standardized rate, 1990–2017	2017 age-standardized PAF	Percentage change in age-standardized PAF, 1990–2017	2017 age-standardized rate per 100,000 people	Percentage change in age-standardized rate, 1990–2017	2017 age-standardized PAF	Percentage change in age-standardized PAF, 1990–2017
**Cardiovascular diseases**	38.9 (24.1, 56.2)	−5.3% (−14.5, 9.9)	16.7% (10.3, 24.1)	36.0% (23.5, 57.6)*	992.4 (649.7, 1,363.9)	2.2% (−9.6, 21.1)	21.6% (14.0, 29.7)	43.3% (27.6, 69.0)*
Ischemic heart disease	20.6 (12.4, 30.3)	−11.3% (−18.2, 0.0) *	17.6% (10.6, 25.9)	26.6% (17.4, 42.3)*	483.4 (309.3, 679.3)	−2.9% (−11.7, 12.0)	22.7% (14.5, 31.9)	34.3% (22.3, 54.2)*
Stroke	13.2 (8.0, 18.9)	−3.7% (−15.1, 15.6)	16.4% (10.0, 23.5)	44.6% (28.0, 73.4)*	406.8 (262.6, 557.3)	4.7% (−9.5, 27.5)	24.5% (15.9, 33.6)	51.2% (31.7, 83.0)*
Ischemic stroke	4.1 (2.3, 6.3)	−13.4% (−20.8, −1.4)*	11.2% (6.2, 17.1)	32.1% (21.8, 50.7)*	124.3 (75.8, 179.9)	4.3% (−6.1, 20.7)	17.7% (10.9, 25.2)	40.8% (28.1, 61.4)*
Intracerebral hemorrhage	7.6 (4.7, 11.2)	4.5% (−9.8, 28.3)	20.0% (12.2, 29.0)	48.6% (29.2, 82.3)*	228.8 (146.2, 315.5)	8.4% (−8.1, 35.1)	28.6% (18.2, 39.2)	56.8% (34.4, 94.9)*
Subarachnoid hemorrhage	1.5 (1.0, 2.0)	−12.2% (−28.9, 13.6)	25.7% (17.0, 35.3)	64.3% (35.9, 116.3)*	53.8 (36.6, 71.8)	−7.6% (−24.9, 18.8)	34.9% (24.1, 45.8)	70.8% (41.1, 122.5)*
Hypertensive heart disease	4.3 (2.2, 6.9)	22.8% (−3.5, 58.3)	34.8% (19.1, 54.5)	52.0% (28.5, 92.4)*	85.1 (53.0, 120.8)	19.6% (−2.8, 54.1)	40.7% (26.3, 56.7)	57.3% (32.5, 100.8)*
Atrial fibrillation and flutter	0.8 (0.5, 1.3)	28.0% (16.8, 46.6)*	20.7% (11.5, 32.2)	26.2% (16.6, 43.8)*	17.0 (9.3, 26.9)	27.0% (16.6, 45.1)*	21.8% (12.6, 33.3)	30.3% (20.3, 48.6)*
**Diabetes and kidney diseases**	9.6 (6.6, 13.1)	70.2% (50.3, 100.3)*	28.6% (19.6, 38.8)	59.7% (41.4, 87.4)*	496.7 (347.2, 679.6)	74.6% (52.7, 107.5)*	38.3% (28.4, 48.7)	65.1% (44.4, 96.0)*
Diabetes mellitus	5.3 (3.7, 7.0)	73.5% (55.9, 99.9)*	30.2% (21.3, 39.8)	55.5% (39.8, 79.2)*	379.4 (261.2, 526.4)	80.4% (58.8, 113.4)*	45.1% (34.1, 56.1)	54.3% (36.2, 81.0)*
Diabetes mellitus type 2	5.3 (3.7, 7.0)	73.5% (55.9, 99.9)*	40.2% (28.5, 52.8)	31.8% (19.1, 51.9)*	379.4 (261.2, 526.4)	80.4% (58.8, 113.4)*	53.4% (40.3, 65.8)	40.7% (24.1, 66.5)*
Chronic kidney disease	4.3 (2.6, 6.3)	66.3% (43.2, 103.6)*	27.0% (16.3, 39.8)	61.8% (39.8, 97.6)*	117.3 (74.6, 165.3)	58.4% (36.5, 93.7)*	26.0% (16.5, 36.8)	73.2% (49.5, 111.6)*
Chronic kidney disease due to diabetes mellitus type 2	1.4 (0.7, 2.3)	94.1% (63.8, 144.1)*	30.5% (15.4, 49.8)	63.6% (38.1, 104.7)*	34.8 (17.1, 54.6)	82.0% (54.3, 126.8)*	34.6% (17.2, 53.3)	66.2% (40.9, 106.5)*
Chronic kidney disease due to hypertension	1.3 (0.6, 2.4)	63.6% (41.7, 104.2)*	28.3% (12.3, 50.4)	53.2% (32.6, 91.3)*	28.9 (14.5, 46.9)	62.5% (38.9, 101.6)*	31.2% (15.6, 49.8)	60.7% (38.1, 98.5)*
Chronic kidney disease due to glomerulonephritis	0.7 (0.3, 1.1)	44.4% (26.8, 73.0)*	28.2% (13.4, 45.4)	56.4% (38.2, 87.3)*	20.2 (8.6, 33.6)	45.6% (26.3, 75.4)*	24.1% (10.4, 39.3)	70.5% (48.3, 106.5)*
Chronic kidney disease due to other and unspecified causes	0.9 (0.4, 1.5)	54.7% (34.4, 88.7)*	27.2% (12.9, 42.7)	68.7% (46.8, 106.2)*	33.3 (15.6, 53.6)	43.4% (24.0, 74.9)*	24.0% (11.3, 38.3)	78.4% (54.5, 118.0)*
**Neoplasms**	5.8 (3.3, 8.9)	23.6% (10.6, 44.7)*	4.8% (2.7, 7.3)	45.3% (30.0, 69.8)*	133.4 (76.5, 205.7)	25.4% (10.4, 50.1)*	4.6% (2.7, 7.1)	54.8% (36.0, 85.2)*
Esophageal cancer	1.0 (0.3, 1.9)	16.6% (−4.4, 52.7)	18.5% (6.2, 35.0)	64.1% (35.4, 113.3)*	23.2 (7.7, 43.1)	13.2% (−7.4, 49.0)	19.4% (6.3, 35.9)	69.8% (40.5, 122.0)*
Colon and rectum cancer	0.9 (0.5, 1.5)	14.5% (5.6, 29.5)*	8.1% (4.4, 12.5)	32.3% (21.9, 50.6)*	20.2 (11.3, 30.9)	17.2% (7.0, 34.0)*	8.6% (4.8, 13.1)	37.0% (25.0, 58.9)*
Liver cancer	1.2 (0.5, 2.3)	61.8% (33.5, 121.3)*	11.9% (4.7, 22.9)	71.0% (42.3, 131.7)*	31.6 (12.6, 61.0)	57.1% (26.7, 123.7)*	12.4% (4.9, 24.0)	84.1% (50.2, 159.0)*
Liver cancer due to hepatitis B	0.5 (0.2, 1.0)	71.2% (33.3, 167.9)*	12.2% (4.5, 24.7)	99.0% (57.1, 204.4)*	14.5 (5.2, 28.8)	61.9% (24.5, 162.3)*	12.5% (4.7, 25.2)	108.7% (62.6, 229.5)*
Liver cancer due to hepatitis C	0.4 (0.2, 0.7)	55.2% (32.9, 100.7)*	13.2% (5.2, 24.3)	55.6% (34.4, 98.5)*	8.6 (3.5, 16.1)	52.0% (28.1, 99.2)*	14.1% (5.7, 25.8)	65.0% (40.2, 115.1)*
Liver cancer due to alcohol use	0.2 (0.1, 0.5)	52.1% (31.3, 90.7)*	14.9% (6.0, 27.9)	51.1% (32.7, 86.4)*	5.7 (2.3, 11.0)	50.5% (28.4, 92.6)*	15.3% (6.2, 28.9)	58.0% (36.8, 99.6)*
Liver cancer due to other causes	0.1 (0.0, 0.2)	70.7% (39.1, 144.9)*	12.4% (4.8, 23.1)	85.6% (50.9, 164.1)*	2.7 (1.0, 5.0)	63.5% (30.6, 142.5)*	12.6% (4.9, 23.5)	98.0% (58.6, 191.9)*
Gallbladder and biliary tract cancer	0.3 (0.2, 0.6)	−6.1% (−15.6, 6.3)	15.4% (8.1, 25.1)	17.2% (7.1, 35.4)*	6.9 (3.7, 11.1)	−3.0% (−13.4, 10.8)	16.0% (8.6, 25.8)	23.8% (12.6, 45.3)*
Pancreatic cancer	0.3 (0.1, 0.6)	36.9% (26.4, 54.6)*	6.2% (2.5, 11.4)	24.0% (14.4, 39.9)**	7.1 (2.8, 13.4)	37.7% (27.2, 55.1)*	6.3% (2.5, 11.9)	28.6% (18.8, 45.0)*
Breast cancer	0.5 (0.2, 0.9)	33.4% (7.6, 100.1)*	6.5% (2.7, 11.8)	50.9% (22.2, 124.9)*	9.9 (3.2, 18.5)	50.5% (8.9, 199.2)*	4.6% (1.5, 8.6)	65.6% (21.2, 230.3)*
Uterine cancer	0.4 (0.3, 0.6)	−2.1% (−11.2, 10.4)	38.7% (26.7, 51.7)	34.8% (23.0, 52.3)*	10.3 (7.1, 13.7)	3.7% (−7.3, 18.6)	39.3% (27.2, 52.1)	40.6% (26.8, 60.4)*
Ovarian cancer	0.1 (0.0, 0.2)	—	3.2% (0.0, 7.2)	—	1.8 (0.0, 4.1)	—	3.2% (0.0, 7.1)	—
Kidney cancer	0.3 (0.2, 0.5)	28.1% (17.0, 45.6)*	19.4% (11.5, 28.6)	22.6% (13.9, 37.7)*	7.6 (4.5, 11.2)	22.8% (11.0, 40.8)*	18.5% (11.0, 27.2)	27.3% (16.4, 45.2)*
Thyroid cancer	0.1 (0.0, 0.1)	31.6% (15.9, 53.1)*	10.1% (4.9, 16.8)	37.6% (21.9, 60.4)*	1.4 (0.7, 2.3)	41.7% (23.7, 66.5)*	10.1% (5.0, 16.8)	45.1% (27.9, 71.9)*
Non-Hodgkin lymphoma	0.2 (0.1, 0.3)	32.2% (21.8, 51.2)*	5.3% (2.2, 9.3)	32.8% (21.3, 53.6)*	4.1 (1.8, 7.3)	31.8% (19.7, 51.1)*	4.6% (2.0, 8.1)	39.5% (23.7, 66.4)*
Multiple myeloma	0.1 (0.0, 0.2)	33.6% (19.4, 54.8)*	7.1% (3.1, 12.4)	31.9% (20.0, 51.5)*	2.1 (0.9, 3.6)	35.3% (21.2, 57.1)*	7.2% (3.2, 12.5)	35.3% (23.3, 55.6)*
Leukemia	0.3 (0.1, 0.5)	11.7% (0.9, 29.2)*	6.3% (3.1, 10.6)	43.9% (28.6, 71.7)*	7.2 (3.6, 12.0)	12.9% (0.3, 32.9)*	4.6% (2.3, 7.6)	61.6% (38.4, 108.6)*
Acute lymphoid leukemia	0.03 (0.01, 0.05)	48.1% (28.1, 72.1)*	4.1% (2.0, 7.0)	50.5% (28.9, 99.0)*	1.0 (0.5, 1.7)	55.2% (31.3, 85.7)*	2.7% (1.3, 4.5)	65.9% (35.7, 135.9)*
Chronic lymphoid leukemia	0.04 (0.02, 0.07)	0.8% (−7.1, 14.8)	8.7% (4.3, 14.2)	23.8% (15.0, 40.4)*	0.8 (0.4, 1.2)	2.3% (−5.6, 15.4)	8.4% (4.2, 13.8)	25.3% (16.5, 40.9)*
Acute myeloid leukemia	0.1 (0.0, 0.1)	44.3% (25.1, 65.9)*	7.0% (3.4, 11.5)	30.6% (17.1, 56.9)*	2.3 (1.1, 3.7)	41.1% (23.8, 64.0)*	5.5% (2.7, 9.0)	35.6% (15.7, 80.2)*
Chronic myeloid leukemia	0.02 (0.01, 0.04)	−38.8% (−43.8, −30.4)*	6.9% (3.4, 11.6)	18.3% (10.0, 32.8)*	0.5 (0.3, 0.9)	−37.5% (−43.5, −27.6)*	6.5% (3.2, 10.9)	22.1% (12.5, 40.0)*
Other leukemia	0.1 (0.1, 0.2)	6.1% (−6.5, 27.5)	5.9% (2.8, 10.0)	66.8% (44.6, 107.8)*	2.7 (1.3, 4.5)	4.5% (−12.0, 31.7)	4.3% (2.1, 7.3)	99.9% (62.7, 174.9)*
**Musculoskeletal disorders**	—	—	—	—	84.1 (44.7, 137.2)	38.1% (24.5, 60.1)*	4.9% (2.9, 7.4)	43.0% (28.9, 65.4)*
Osteoarthritis	—	—	—	—	25.1 (9.9, 53.8)	55.6% (39.9, 84.0)*	21.1% (11.9, 31.5)	42.0% (28.1, 67.8)*
Low back pain	—	—	—	—	53.8 (28.9, 91.1)	29.9% (17.8, 50.3)*	6.6% (3.9, 9.8)	43.0% (29.4, 65.6)*
Gout	—	—	—	—	5.2 (2.7, 8.7)	54.8% (38.3, 82.8)*	32.4% (18.7, 49.2)	44.4% (28.8, 70.0)*
**Neurological disorders**	4.5 (1.7, 8.4)	21.1% (10.5, 45.3)*	10.4% (3.9, 19.6)	26.7% (15.6, 51.8)*	52.5 (21.1, 98.0)	21.8% (10.7, 45.2)*	3.7% (1.5, 7.1)	26.4% (14.5, 50.9)*
Alzheimer’s disease and other dementias	4.5 (1.7, 8.4)	21.1% (10.5, 45.3)*	12.7% (4.8, 23.8)	26.1% (15.2, 51.2)*	52.5 (21.1, 98.0)	21.8% (10.7, 45.2)*	12.7% (5.2, 23.8)	29.7% (17.8, 55.2)*
**Chronic respiratory diseases**	0.9 (0.5, 1.6)	−20.6% (−35.6, 5.4)	1.8% (0.9, 3.1)	38.3% (12.7, 84.0)*	44.4 (25.8, 69.5)	−1.3% (−19.2, 23.5)	3.1% (1.8, 4.8)	59.6% (32.4, 99.4)*
Asthma	0.9 (0.5, 1.6)	−20.6% (−35.6, 5.4)	14.5% (7.7, 23.1)	83.1% (49.6, 146.1)*	44.4 (25.8, 69.5)	−1.3% (−19.2, 23.5)	15.1% (9.1, 22.7)	80.2% (50.0, 127.9)*
**Digestive diseases**	0.5 (0.3, 0.7)	−5.0% (−15.3, 11.5)	1.5% (0.9, 2.2)	31.1% (16.9, 53.3)*	7.7 (4.9, 10.9)	−6.3% (−18.4, 13.3)	0.7% (0.5, 1.0)	24.6% (8.0, 51.7)*
Gallbladder and biliary diseases	0.5 (0.3, 0.7)	−5.0% (−15.3, 11.5)	31.0% (19.5, 44.1)	34.7% (21.9, 55.7)*	7.7 (4.9, 10.9)	−6.3% (−18.4, 13.3)	30.0% (19.4, 42.2)	44.2% (26.7, 71.5)*
**Sense organ diseases**	—	—	—	—	5.8 (2.6, 10.7)	44.0% (27.7, 71.4)*	0.7% (0.3, 1.2)	46.8% (30.3, 74.7)*
Blindness and vision impairment	—	—	—	—	5.8 (2.6, 10.7)	44.0% (27.7, 71.4)*	1.6% (0.7, 2.8)	50.8% (34.0, 79.2)*
Cataract	—	—	—	—	5.8 (2.6, 10.7)	44.0% (27.7, 71.4)*	5.7% (2.7, 9.6)	51.1% (34.6, 79.7)*

*Changes that are statistically significant.

DALY, disability-adjusted life year; PAF, population attributable fraction.

**Fig 3:**
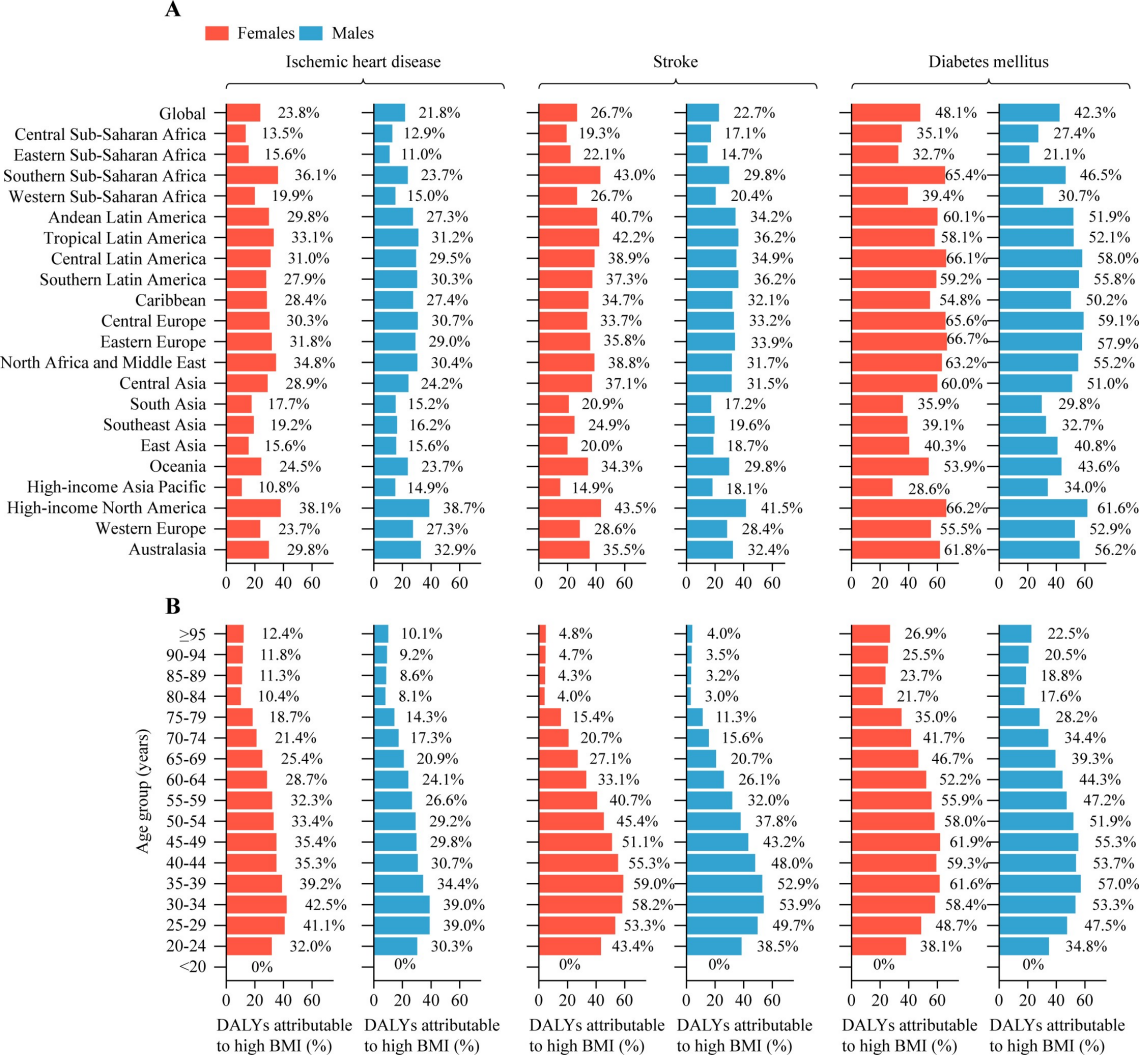
Fraction of ischemic heart disease, stroke, and diabetes mellitus age-standardized DALYs attributable to high BMI by region and by age group for females and males in 2017. (A) By region. (B) By age group. The 3 leading GBD level 3 causes of high-BMI-related DALYs are shown. BMI, body mass index; DALY, disability-adjusted life year; GBD, Global Burden of Disease Study.

For each GBD level 3 cause, global DALYs attributable to high BMI were highest for ischemic heart disease (483.4 [95% UI 309.3, 679.3] per 100,000 people), stroke (406.8 [95% UI 262.6, 557.3] per 100,000 people), diabetes mellitus (379.4 [95% UI 261.2, 526.4] per 100,000 people), chronic kidney disease (117.3 [95% UI 74.6, 165.3] per 100,000 people), hypertensive heart disease (85.1 [95% UI 53.0, 120.8] per 100,000 people), and low back pain (53.8 [95% UI 28.9, 91.1] per 100,000 people; Table 1). Of all ischemic heart disease age-standardized DALYs worldwide, 22.7% (95% UI 14.5%, 31.9%) was attributable to high BMI; the corresponding proportions were 24.5% (95% UI 15.9%, 33.6%) for stroke, 45.1% (95% UI 34.1%, 56.1%) for diabetes mellitus, 26.0% (95% UI 16.5%, 36.8%) for chronic kidney disease, 40.7% (95% UI 26.3%, 56.7%) for hypertensive heart disease, and 6.6% (95% UI 3.9%, 9.8%) for low back pain. For most GBD level 3 causes, the proportional contribution of high BMI to age-standardized DALYs was larger in females than in males, with the exception of colon and rectum cancer, liver cancer, non-Hodgkin lymphoma, and gout (Figs 3 and S3–S10).

There were substantial differences in the attributable proportions of age-standardized DALYs due to high BMI across regions and age groups (Figs 3 and S3–S10). Across the 21 GBD regions, in 2017, high-income North America had the highest attributable proportions of age-standardized DALYs due to high BMI for most GBD level 3 causes in both females (21 of 26 GBD level 3 causes) and males (21 of 23 GBD level 3 causes), whereas high-income Asia Pacific had the lowest attributable proportions for most GBD level 3 causes in females (21 of 26 GBD level 3 causes), and eastern sub-Saharan Africa had the lowest attributable proportions for most GBD level 3 causes in males (21 of 23 GBD level 3 causes). By age group, as shown in Figs 3 and S3–S10, the attributable proportions of age-standardized DALYs due to high BMI varied widely across different causes in both sexes. Notably, high BMI was only associated with asthma DALYs among children aged <20 years and was a protective factor for breast cancer in females in the age groups from 20 to <50 years.

### Relationship between SDI and the impact of high BMI on disease burden

**Fig 4:**
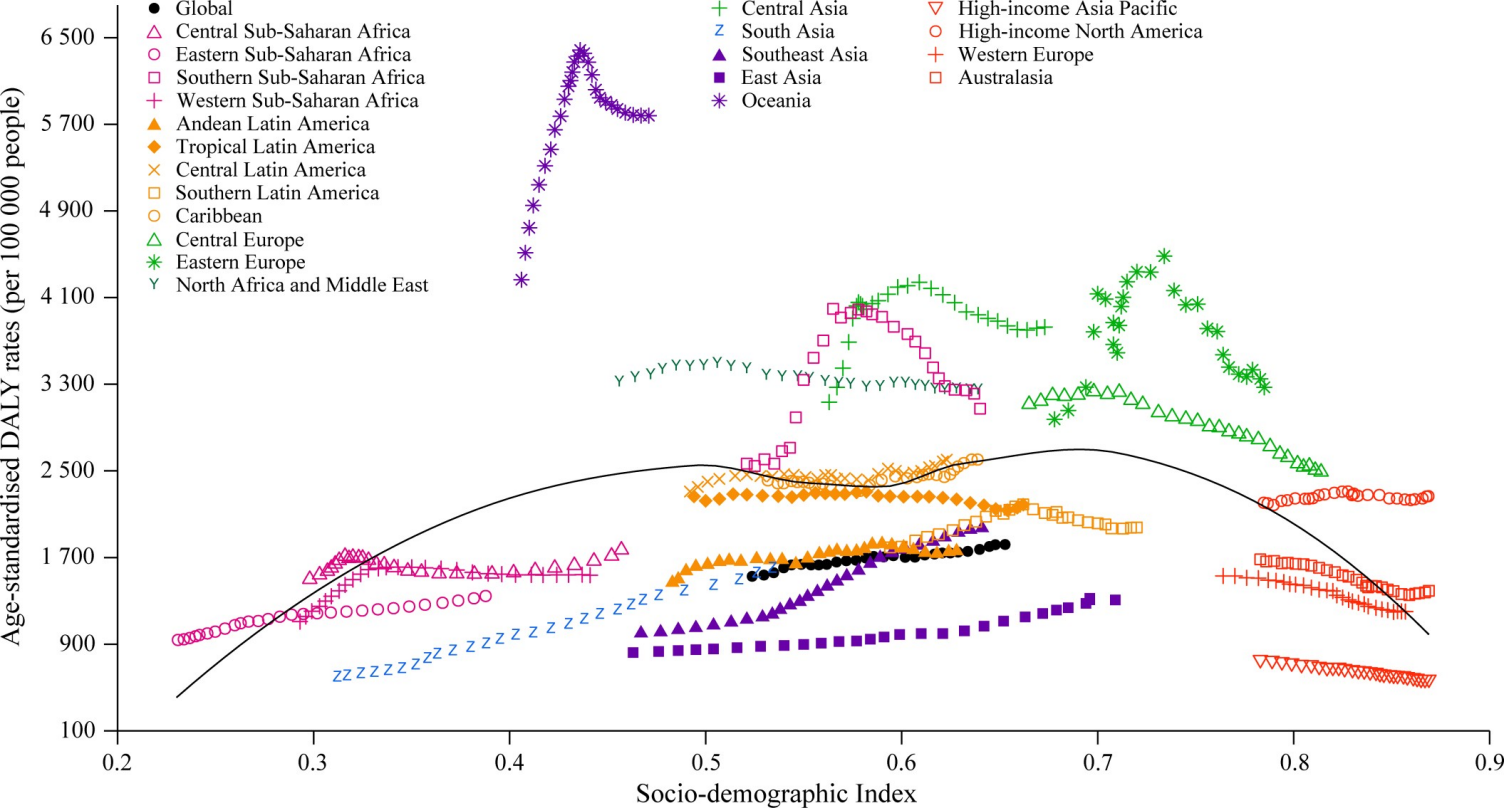
Age-standardized DALY rates attributable to high body mass index across 21 GBD regions by Socio-demographic Index for both sexes combined, 1990–2017. For each region, points from left to right depict estimates from each year from 1990 to 2017. DALY, disability-adjusted life year; GBD, Global Burden of Disease Study.

**Fig 5:**
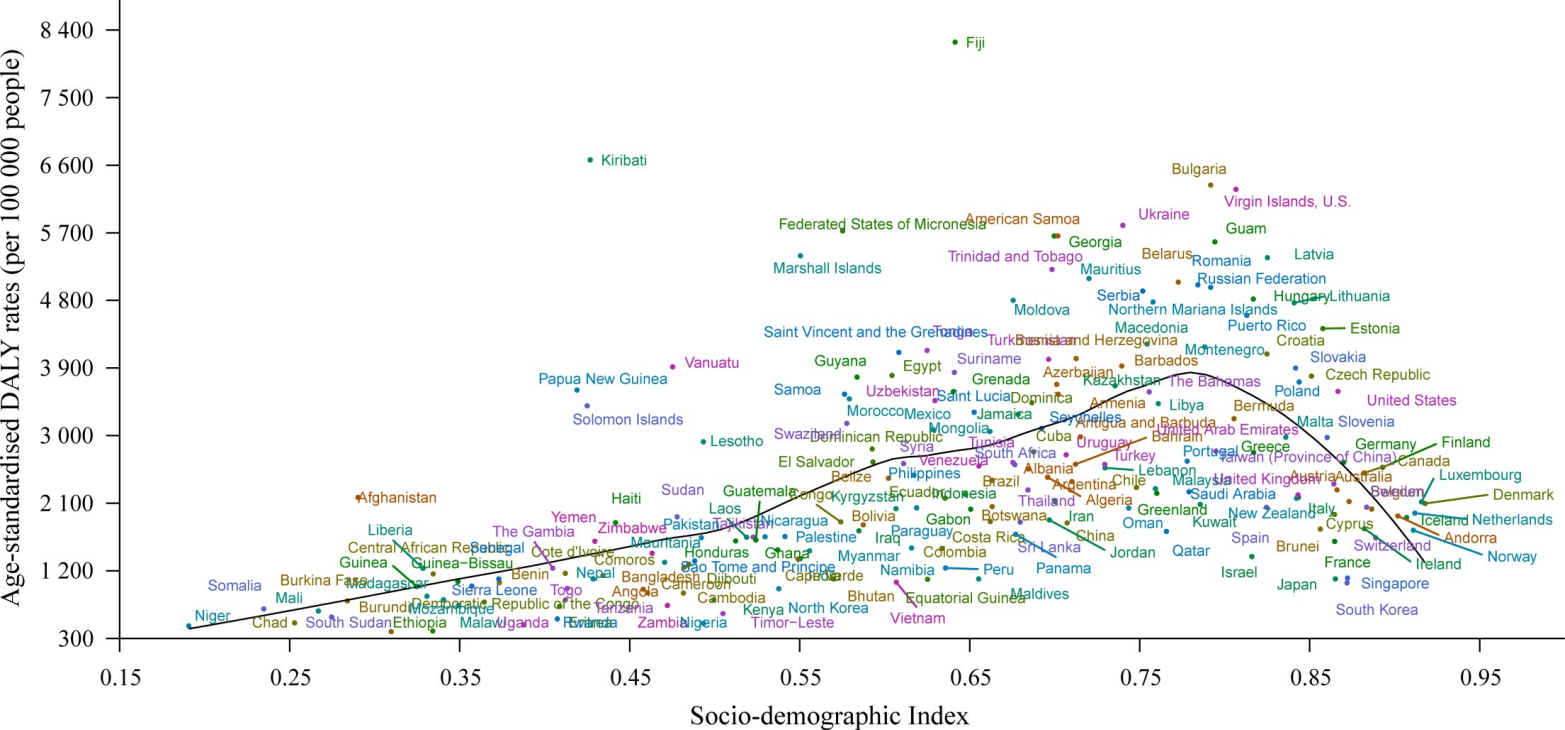
Age-standardized DALY rates attributable to high body mass index across 195 countries and territories by Socio-demographic Index for both sexes combined in 2017. DALY, disability-adjusted life year.

In 2017, the lowest age-standardized rates of high-BMI-related deaths and DALYs were observed in countries in the low-SDI quintile (38.8 [95% UI 19.8, 63.1] deaths per 100,000 people and 1,240.2 [95% UI 672.0, 1,912.3] DALYs per 100,000 people) and high-SDI quintile (44.9 [95% UI 29.3, 62.2] deaths per 100,000 people and 1,462.3 [95% UI 1,007.4, 1,963.7] DALYs per 100,000 people; S1 Table). Fig 4 shows the changes in age-standardized DALY rates across SDI by region, from 1990 to 2017. Of the 4 regions with the highest SDI, 3 exhibited a decline in age-standardized rate of high-BMI-related DALYs, whereas the DALY rates in high-income North America remained stable between 1990 and 2017. Additionally, all 4 regions with the highest SDI experienced a decline in age-standardized rate of high-BMI-related deaths during the study period (S11 Fig). In contrast, from 1990 to 2017, all 4 regions with the lowest SDI experienced an increase in age-standardized rates of high-BMI-related deaths and DALYs (Figs 4 and S11). Fig 5 shows the association between age-standardized DALY rate and SDI across countries in 2017. Across countries, as SDI increases, age-standardized DALY rate increases until SDI is about 0.78, but then decreases with higher SDI. Based solely on SDI, the age-standardized DALY rates were much higher than expected in Kiribati and Fiji.

## Discussion

Our findings showed that, globally, from 1990 to 2017, both deaths and DALYs attributable to high BMI have more than doubled in both sexes. However, after age-standardizing the high-BMI-related DALY rates, only a slight increase was noted in both sexes. The marked increase in the number of global deaths and DALYs attributable to high BMI can be partially explained by taking into account the aging and growth of the population. Moreover, our study found that the global deaths and DALYs attributable to high BMI were higher in females than in males aged ≥70 years old, but lower in females than in males aged <70 years old. The reason of this phenomenon is not completely understood. However, the worldwide prevalence rate of overweight and obesity was found to be significantly higher in older females than in older males [10,19]. The sex differences with respect to high-BMI-related DALYs should be addressed by designing and implementing ad hoc strategies, which are urgently needed, since, as of today, policies and programs developed to target specific disorders, such as those generated by high BMI, do not generally adopt a sex-based lens [20].

Despite clinical and scientific achievements that have led to a better understanding of obesity pathogenesis and better management of patients with obesity, high BMI has not shown a declining pattern but rather an upward one in the last decades [10,19]. This increase in the prevalence rate of obesity and its generated burden of disease warrants surveillance and monitoring of BMI on a regular basis as well as the analysis and identification of the determinants of such an increase, in order to design and implement appropriate evidence-based public health measures and interventions, and assess and eventually revise their effectiveness.

As previously reported, cardiovascular disease, diabetes, and kidney diseases were among the leading causes of high-BMI-related death and DALYs [10]. Notably, among all GBD level 3 causes, low back pain ranked as the sixth leading cause of high-BMI-related DALYs. Interventions targeting high BMI could counteract or at least mitigate the burden imposed by low back pain and other related musculoskeletal disorders [21]. In addition, interestingly, high BMI was found to be a protective factor against breast cancer in females aged 20–50 years. This result could be explained with the theory of the “obesity paradox,” which states that obesity may decrease the risk of developing some cancers [22]. Other possible explanations could be methodological flaws, such as improper or incorrect adjustment for confounding factors, and the weakness of BMI as a surrogate biomarker to fully capture body composition.

In terms of SDI, there was a temporal increasing pattern in high-BMI-related disease burden in regions with the lowest SDI, whereas a decreasing -pattern could be noted for regions with the highest SDI, with the exception of high-income North America. The temporal changes in high-BMI-related burden can only partially be explained by taking into account income levels, given the complex, nonlinear relationship between overweight, obesity, and SDI.

Increased food and beverage consumption is the outcome of the interaction among different factors, including changes in agricultural practices, food environment, and food industry, as well as the implementation of various food and drink restrictions and taxation introduced since 2017 to combat weight gain, which have resulted in considerable shifts in dietary patterns. High calorie food; food that is ultra-processed, highly energy-dense, and rich in fat, salt, and glycemic load; and carbonated beverages have become increasingly available and affordable, and such accessibility, together with food marketing and advertising and the widespread presence of transnational beverage and food corporations, is one of the major determinants of the rise of high BMI in the last decades [23–25]. Besides food availability and transformation systems, urbanization, though to a lesser extent, represents another driver of weight gain, together with sedentary lifestyle, low physical activity, and increased time spent watching television or playing videogames [26–28].

However, increases in BMI have been recorded also in rural and underserved areas, which, being resource-limited settings, cannot implement policies for providing access to and distributing healthy food and are generally plagued by undernutrition. In contrast, high-income countries have adequate financial resources to launch programs and initiatives aimed at promoting healthy food (which is generally more expensive than “junk food”) [29]. On the other hand, as previously mentioned, the relationship between SDI and healthy food consumption is multifaceted and nonlinear, with North America being a notable exception to the pattern seen in other high-income countries and being more inclined to follow a “Westernized” diet. Furthermore, other factors, still not well understood, may play a key role, including the availability of poorly effective pharmacological options, and an increased level of stress characterizing current society, which induces individuals to choose and consume junk food (the “comfort food hypothesis”) [30].

As such, the burden of disease imposed by high BMI is particularly dramatic. Public health policy- and decision-makers and other relevant stakeholders should pay attention to this issue and prioritize their agenda, designing and implementing ad hoc, effective policies. Evidence-based interventions should be aimed at empowering and educating individuals, making them aware of the potentially detrimental consequences of their overweight/obesity [31], and promoting physical activity. New information and communication technologies could be exploited for the purposes of both gathering BMI and other relevant information and fostering changes, tracking changes over the time [32].

It is of paramount importance to regularly measure the BMI of individuals starting from childhood. A 2-year longitudinal investigation [33] recruited students aged 13–17 years sampled from the COMPASS study, and stratified them into 4 behavioral pattern clusters: “typical high school athletes,” “inactive high screen-users,” “moderately active substance users,” and “health conscious.” Membership in a behavioral pattern was not able to predict BMI trajectories but only baseline BMI status. This implies that the earlier the behaviorally oriented intervention, the higher the impact of the measure in terms of health gains. Early modifications of unhealthy behavioral risk factors starting from childhood may counteract, or at least mitigate, the increase of overweight and obesity.

The present study provides stakeholders with estimates at the country and global level, covering a span of 28 years, in such a way as to measure the effectiveness of interventions and monitor this effectiveness through time. However, the present study is not without limitations. Data utilized include self-report measures, which, even though corrected, do not incorporate measurement uncertainty. Cutoffs utilized may fail to capture overweight and obesity in some populations, such as those from Asia, or groups at risk, with underlying comorbidities. For some countries, data may be based on information derived from samples not necessarily representative of the entire country/territory under study. Moreover, BMI, despite being a popular metric, cannot distinguish between lean and fat mass, because it is not able to account for bone density and body composition. On the other hand, BMI is based on measures that can be easily collected at the population level.

In summary, high BMI is an important contributor to global disease burden. Researchers should contribute to efforts to counteract the burden generated by high BMI, by better assessing the changing patterns of high BMI on the global scale and devising strategies that can be implemented to foster behavioral changes, whereas public and global health policy- and decision-makers should allocate specific funding and prioritize this crucial topic on their agendas [34–36].

## Supporting information

S1 GATHER Checklist GATHER checklist of information that should be included in reports of global health estimates. (DOCX)

S1 Fig Numbers of all-age deaths and DALYs attributable to high body mass index by sex, 1990–2017. (A) Deaths. (B) DALYs. DALY, disability-adjusted life year.
(TIF)

S2 Fig Age-standardized death and DALY rates attributable to high body mass index by sex, 1990–2017. (A) Deaths. (B) DALYs. DALY, disability-adjusted life year.
(TIF)

S3 Fig Fraction of chronic kidney disease, hypertensive heart disease, and low back pain age-standardized DALYs attributable to high body mass index by region and by age group for females and males in 2017. (A) By region. (B) By age group. The fourth to sixth leading GBD level 3 causes of high-BMI-related DALYs are shown. BMI, body mass index; DALY, disability-adjusted life year; GBD, Global Burden of Disease Study.
(TIF)

S4 Fig Fraction of atrial fibrillation and flutter, esophageal cancer, and colon and rectum cancer age-standardized DALYs attributable to high body mass index by region and by age group for females and males in 2017. (A) By region. (B) By age group. BMI, body mass index; DALY, disability-adjusted life year.
(TIF)

S5 Fig Fraction of liver cancer, gallbladder and biliary tract cancer, and pancreatic cancer age-standardized DALYs attributable to high body mass index by region and by age group for females and males in 2017. (A) By region. (B) By age group. BMI, body mass index; DALY, disability-adjusted life year.
(TIF)

S6 Fig Fraction of breast cancer, uterine cancer, and ovarian cancer age-standardized DALYs attributable to high body mass index by region and by age group for females in 2017. (A) By region. (B) By age group. BMI, body mass index; DALY, disability-adjusted life year.
(TIF)

S7 Fig Fraction of kidney cancer, thyroid cancer, and non-Hodgkin lymphoma age-standardized DALYs attributable to high body mass index by region and by age group for females and males in 2017. (A) By region. (B) By age group. BMI, body mass index; DALY, disability-adjusted life year.
(TIF)

S8 Fig Fraction of multiple myeloma, leukemia, and osteoarthritis age-standardized DALYs attributable to high body mass index by region and by age group for females and males in 2017. (A) By region. (B) By age group. BMI, body mass index; DALY, disability-adjusted life year.
(TIF)

S9 Fig Fraction of gout, Alzheimer’s disease and other dementias, and asthma age-standardized DALYs attributable to high body mass index by region and by age group for females and males in 2017. (A) By region. (B) By age group. BMI, body mass index; DALY, disability-adjusted life year.
(TIF)

S10 Fig Fraction of gallbladder and biliary diseases, and blindness and vision impairment age-standardized DALYs attributable to high body mass index by region and by age group for females and males in 2017. (A) By region. (B) By age group. BMI, body mass index; DALY, disability-adjusted life year.
(TIF)

S11 Fig Age-standardized death rates attributable to high body mass index across 21 GBD regions by Socio-demographic Index for both sexes combined, 1990–2017. For each region, points from left to right depict estimates from each year from 1990 to 2017. DALY, disability-adjusted life year; GBD, Global Burden of Disease Study.
(TIF)

S1 Table Age-standardized deaths and DALYs attributable to high body mass index for both sexes combined in 2017 and percentage change from 1990 to 2017, by location. (DOCX)

## Abbreviations

BMI: body mass index
DALY: disability-adjusted life year
GBD: Global Burden of Disease Study
SDI: Socio-demographic Index
UI: uncertainty interval
