# Supplementary figures and images for: The global burden of disease attributable to high body mass index in 195 countries and territories, 1990–2017: An analysis of the Global Burden of Disease Study
Source: PLoS Med. 2020 Jul 28;17(7):e1003198. doi: 10.1371/journal.pmed.1003198 (PMC7386577; doi:10.1371/journal.pmed.1003198)

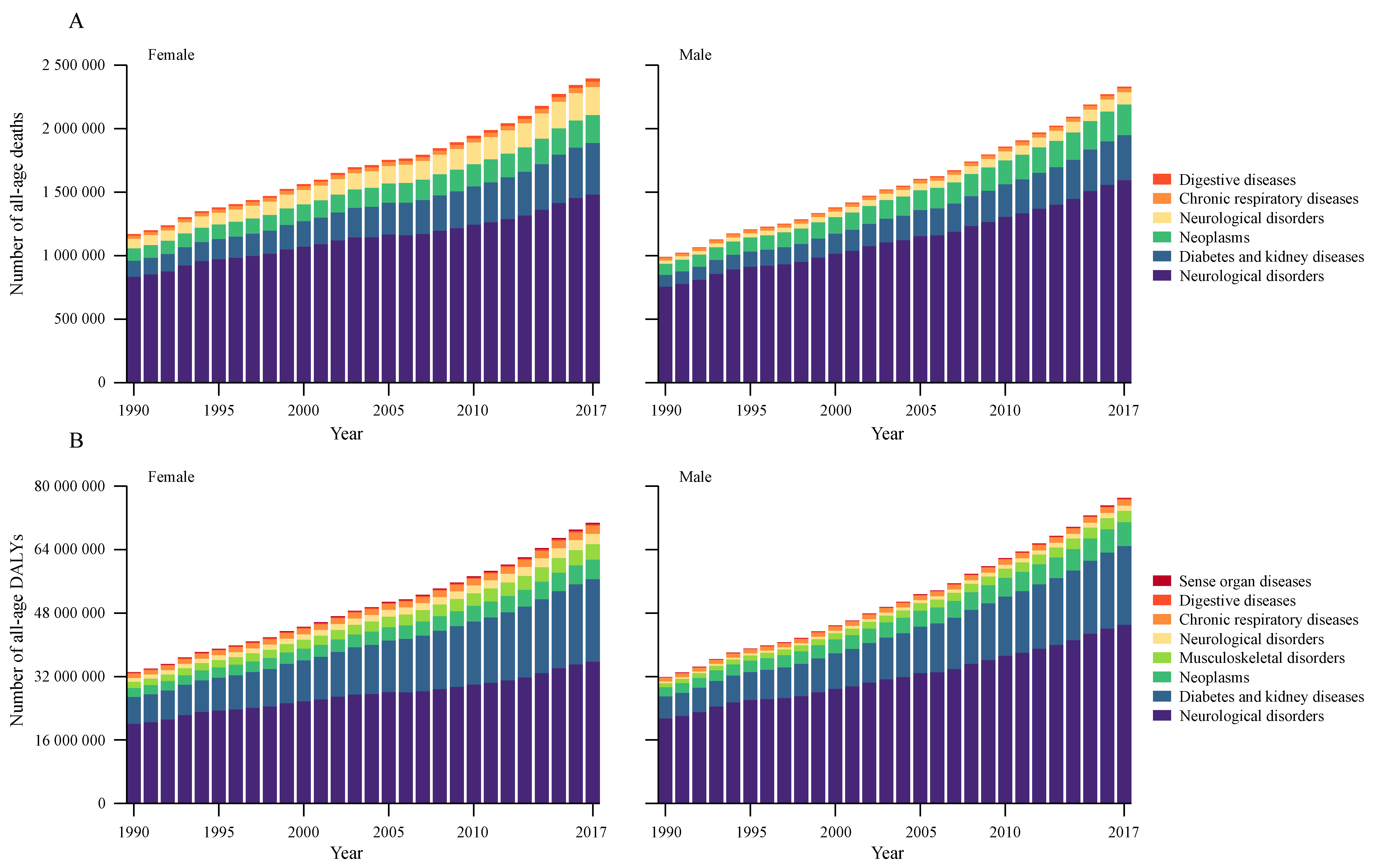

Supplement: S1 Fig — (A) Deaths. (B) DALYs. DALY, disability-adjusted life year. (TIF) [file pmed.1003198.s002.tif]

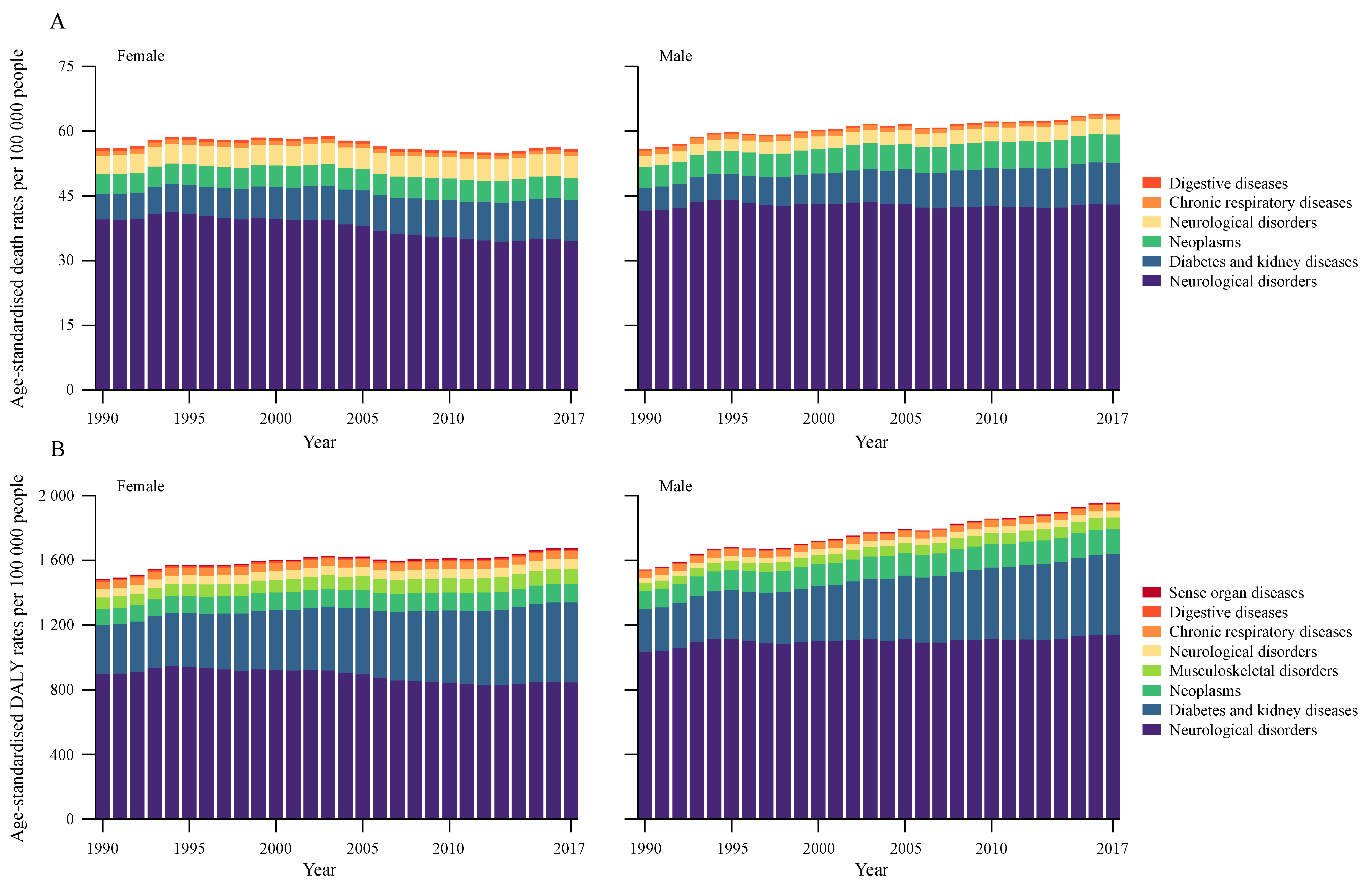

Supplement: S2 Fig — (A) Deaths. (B) DALYs. DALY, disability-adjusted life year. (TIF) [file pmed.1003198.s003.tif]

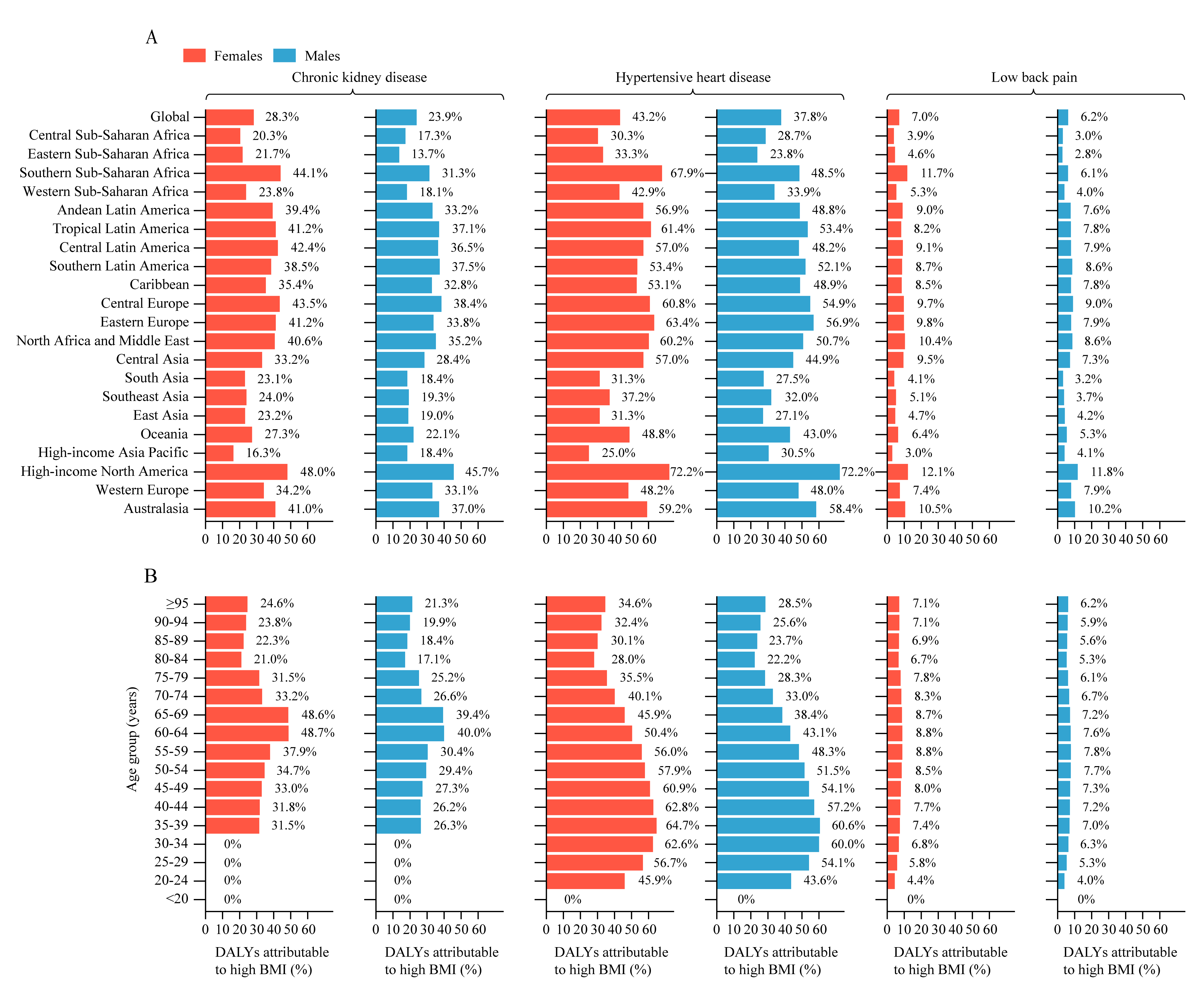

Supplement: S3 Fig — (A) By region. (B) By age group. The fourth to sixth leading GBD level 3 causes of high-BMI-related DALYs are shown. BMI, body mass index; DALY, disability-adjusted life year; GBD, Global Burden of Disease Study. (TIF) [file pmed.1003198.s004.tif]

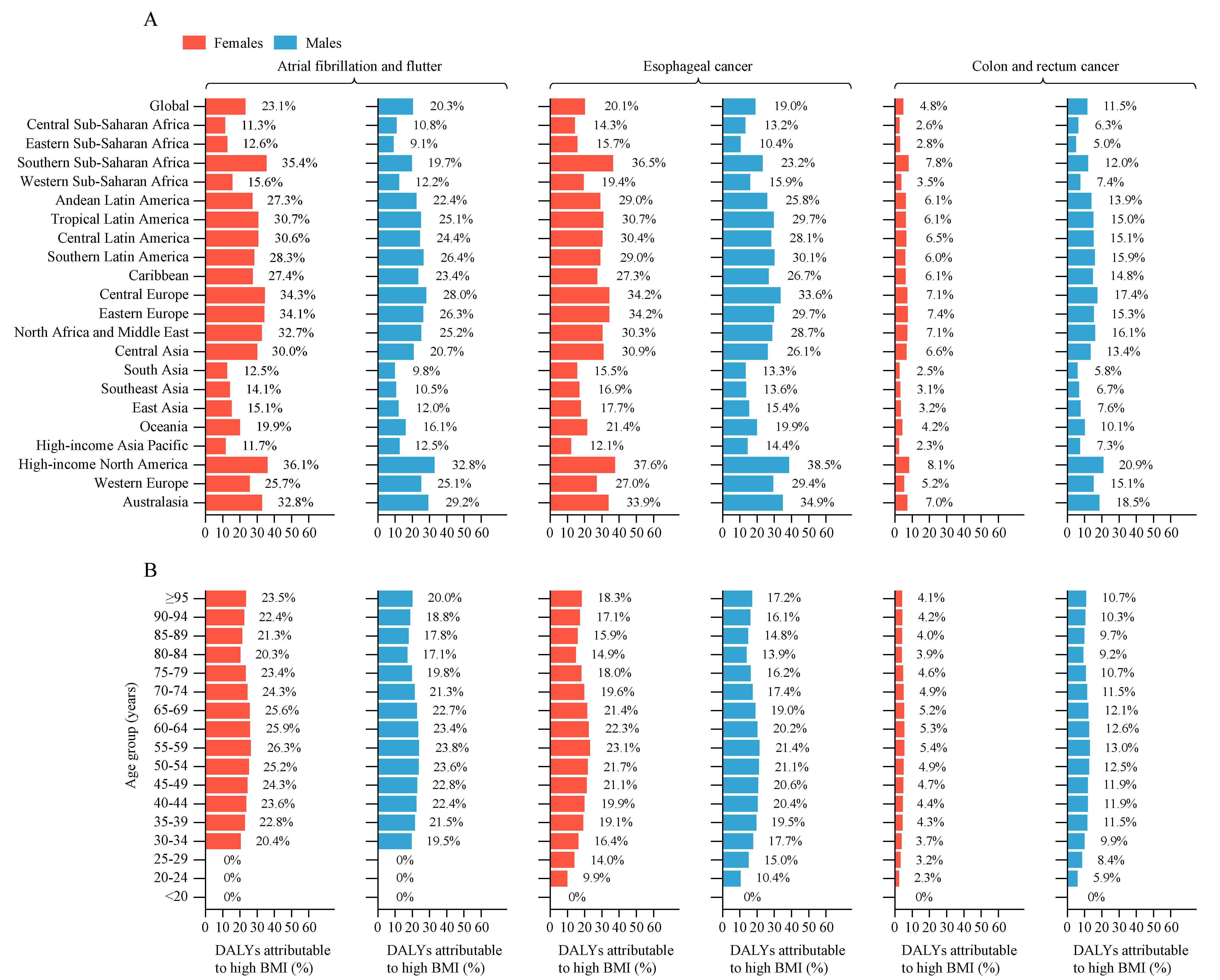

Supplement: S4 Fig — (A) By region. (B) By age group. BMI, body mass index; DALY, disability-adjusted life year. (TIF) [file pmed.1003198.s005.tif]

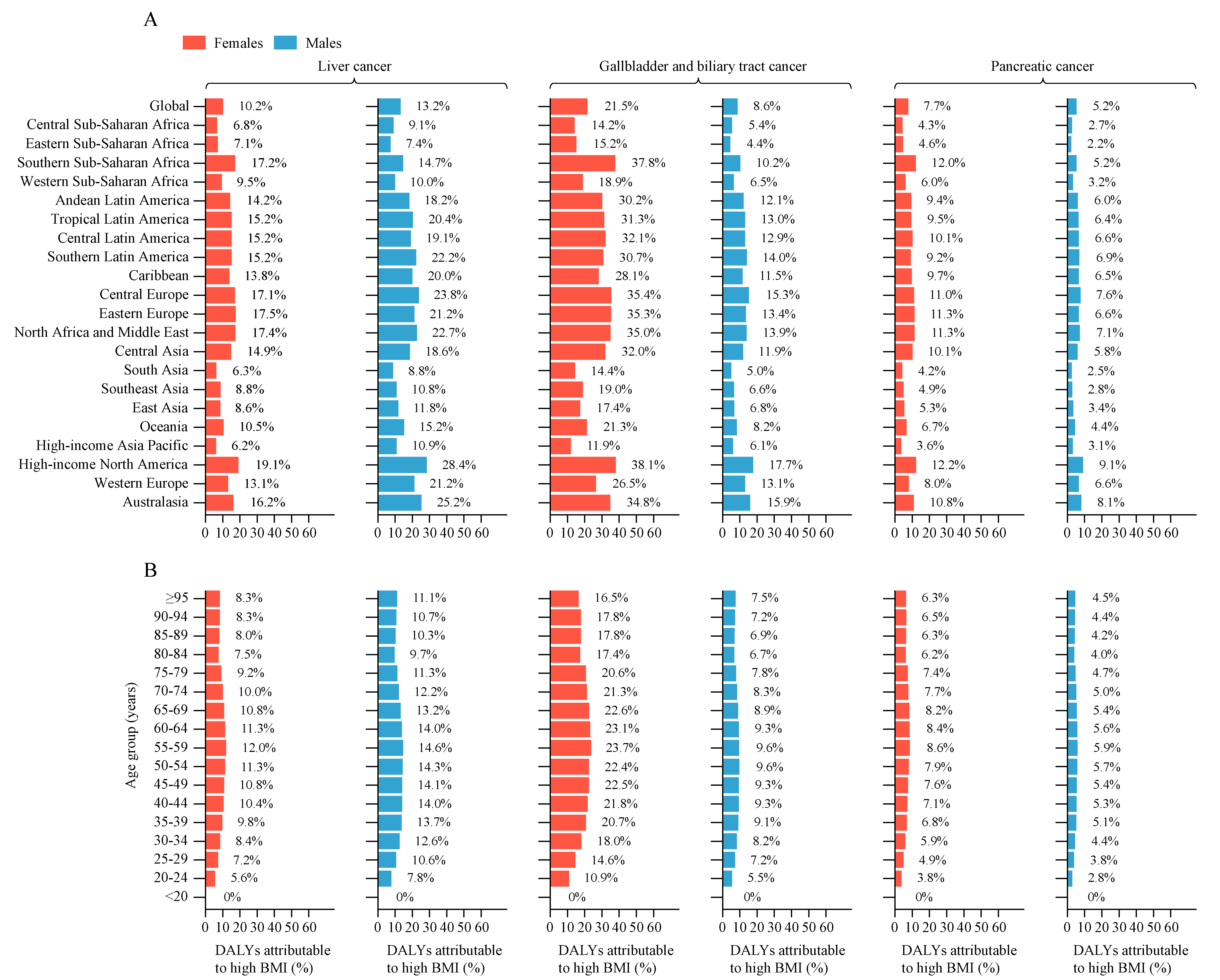

Supplement: S5 Fig — (A) By region. (B) By age group. BMI, body mass index; DALY, disability-adjusted life year. (TIF) [file pmed.1003198.s006.tif]

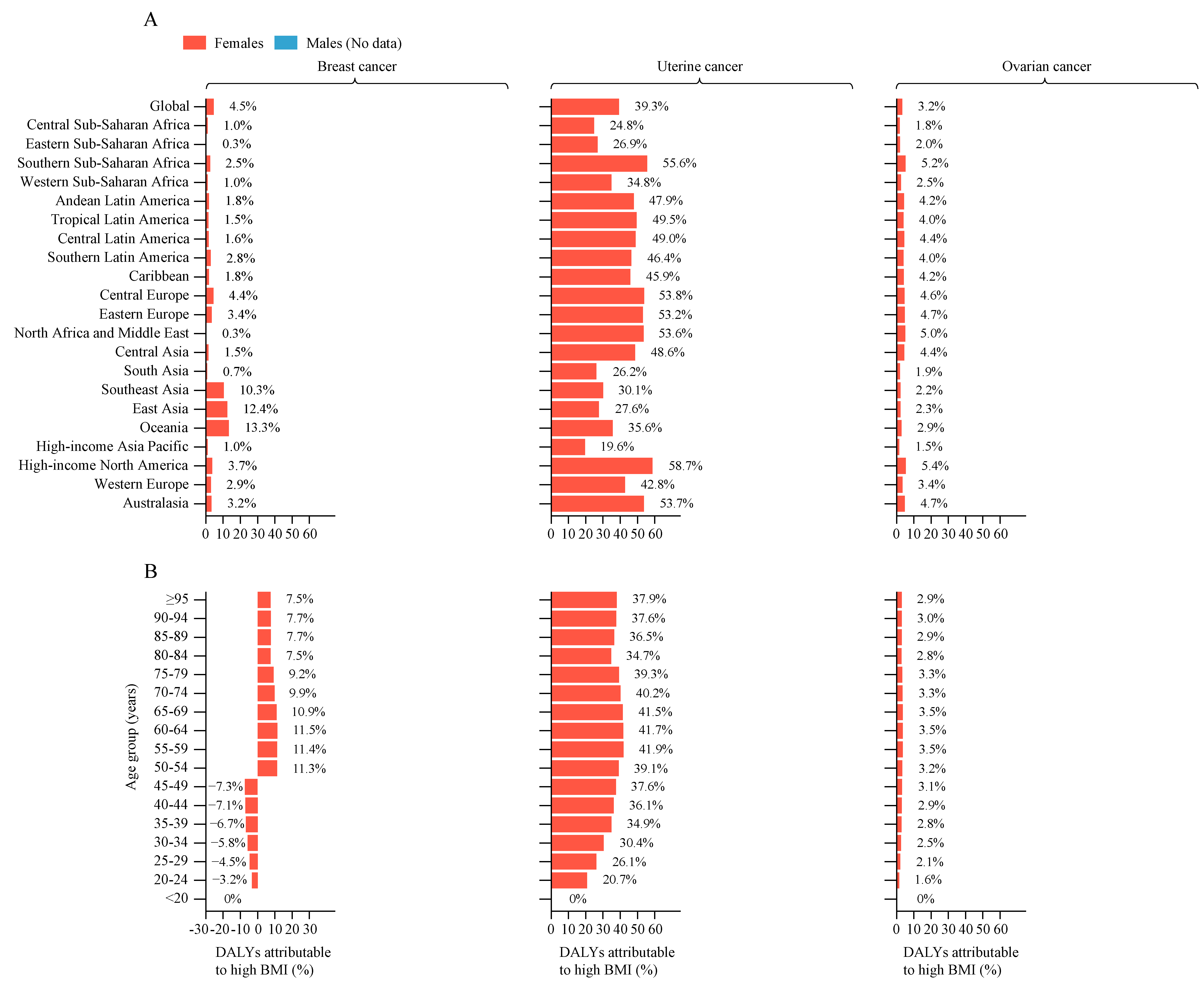

Supplement: S6 Fig — (A) By region. (B) By age group. BMI, body mass index; DALY, disability-adjusted life year. (TIF) [file pmed.1003198.s007.tif]

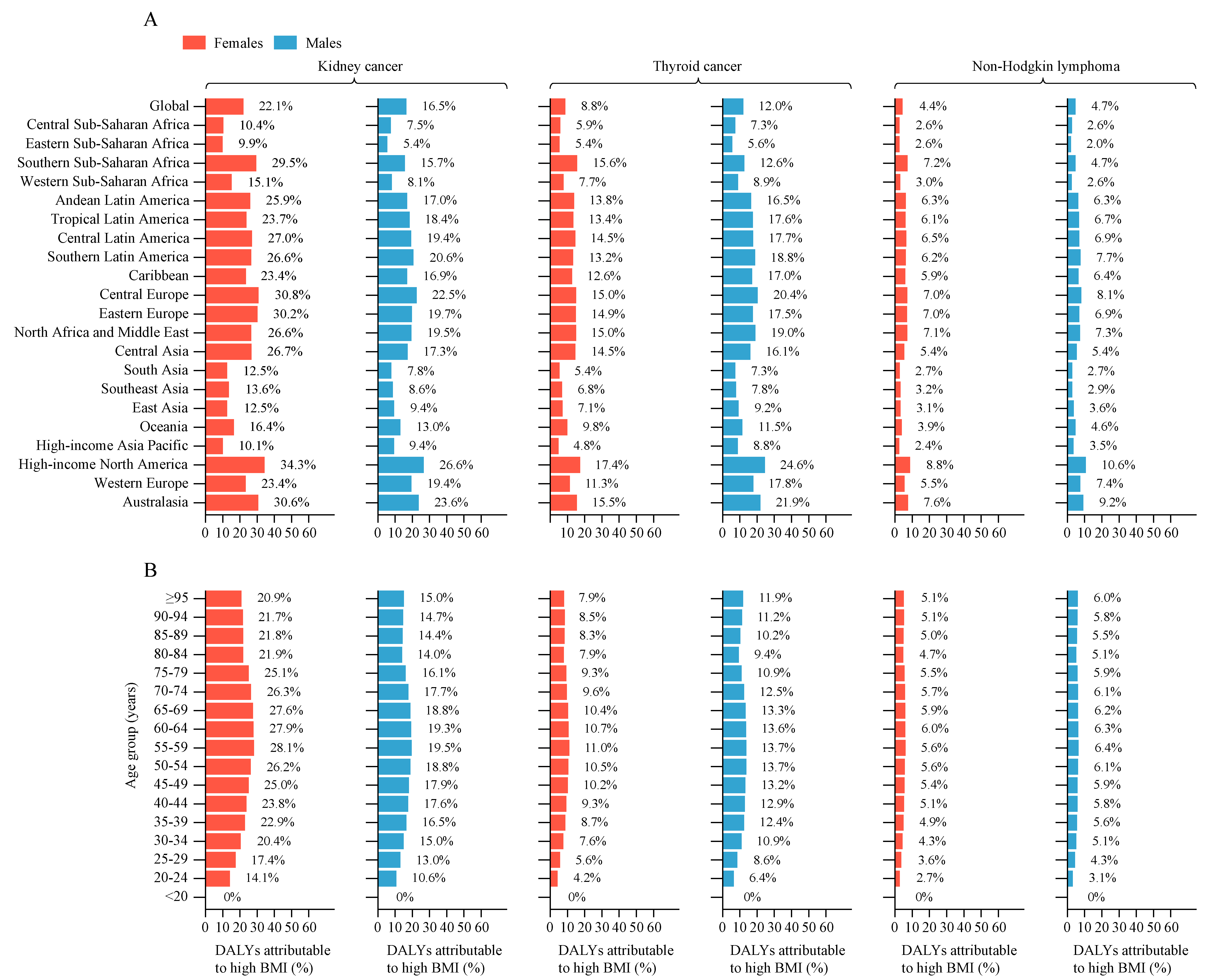

Supplement: S7 Fig — (A) By region. (B) By age group. BMI, body mass index; DALY, disability-adjusted life year. (TIF) [file pmed.1003198.s008.tif]

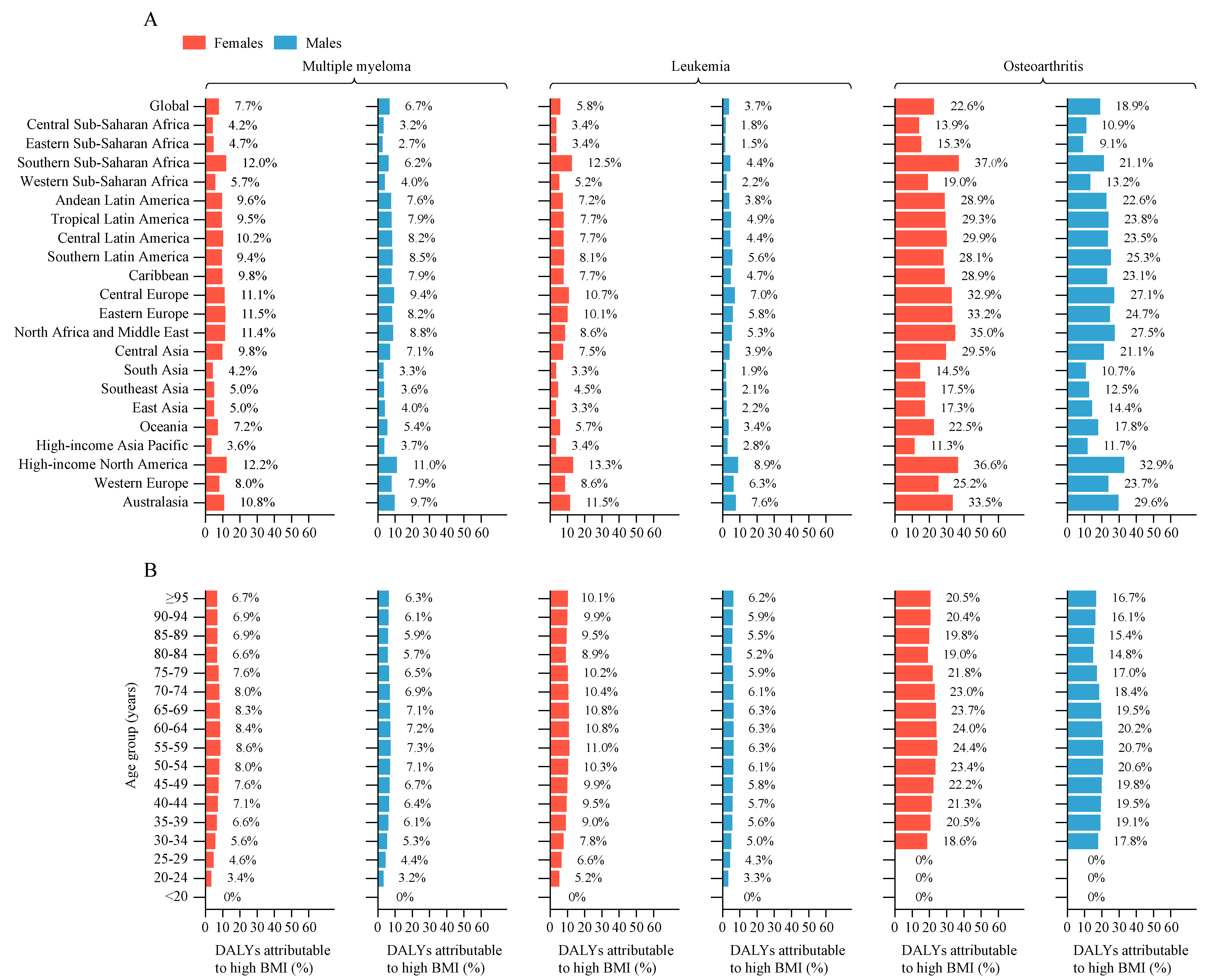

Supplement: S8 Fig — (A) By region. (B) By age group. BMI, body mass index; DALY, disability-adjusted life year. (TIF) [file pmed.1003198.s009.tif]

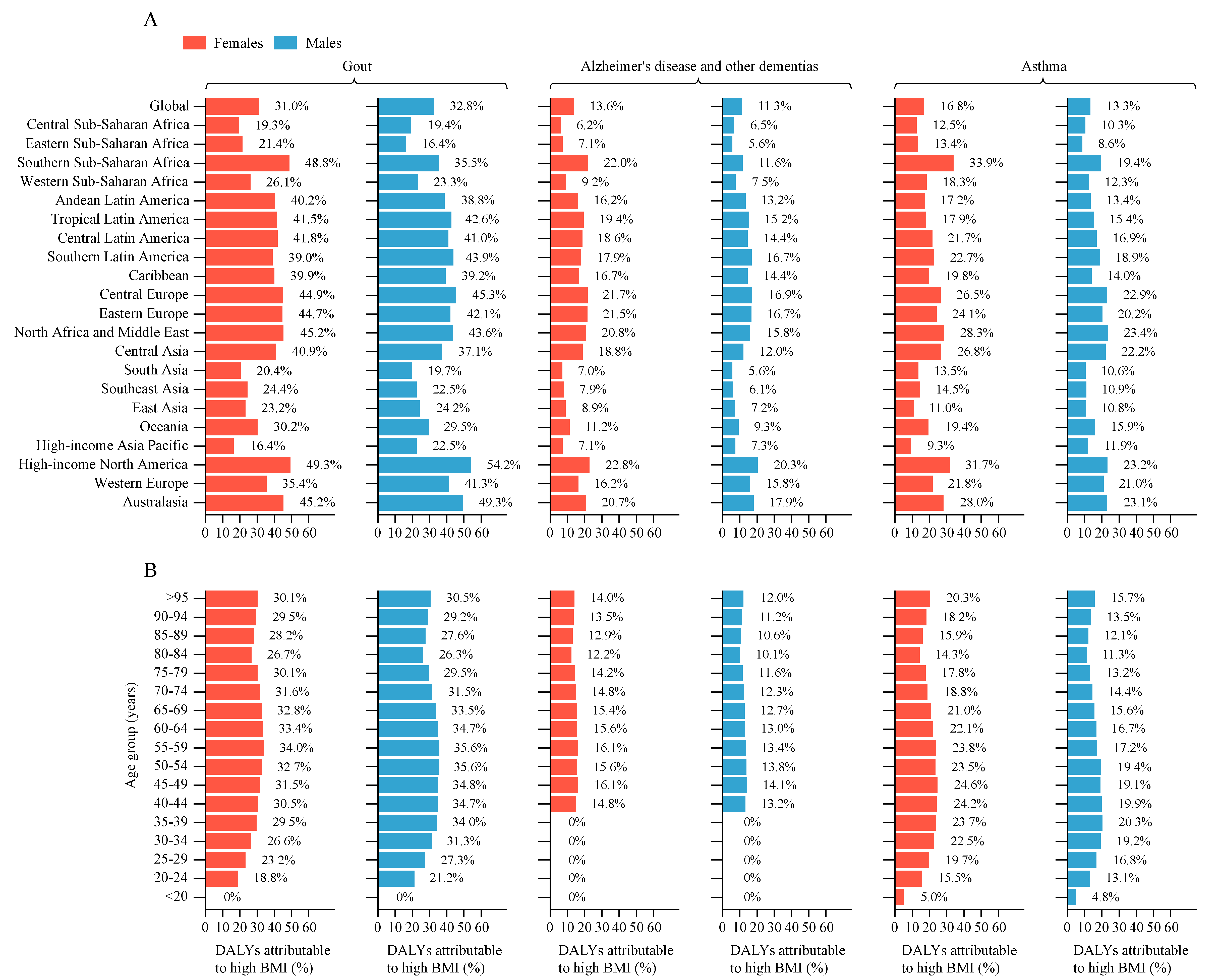

Supplement: S9 Fig — (A) By region. (B) By age group. BMI, body mass index; DALY, disability-adjusted life year. (TIF) [file pmed.1003198.s010.tif]

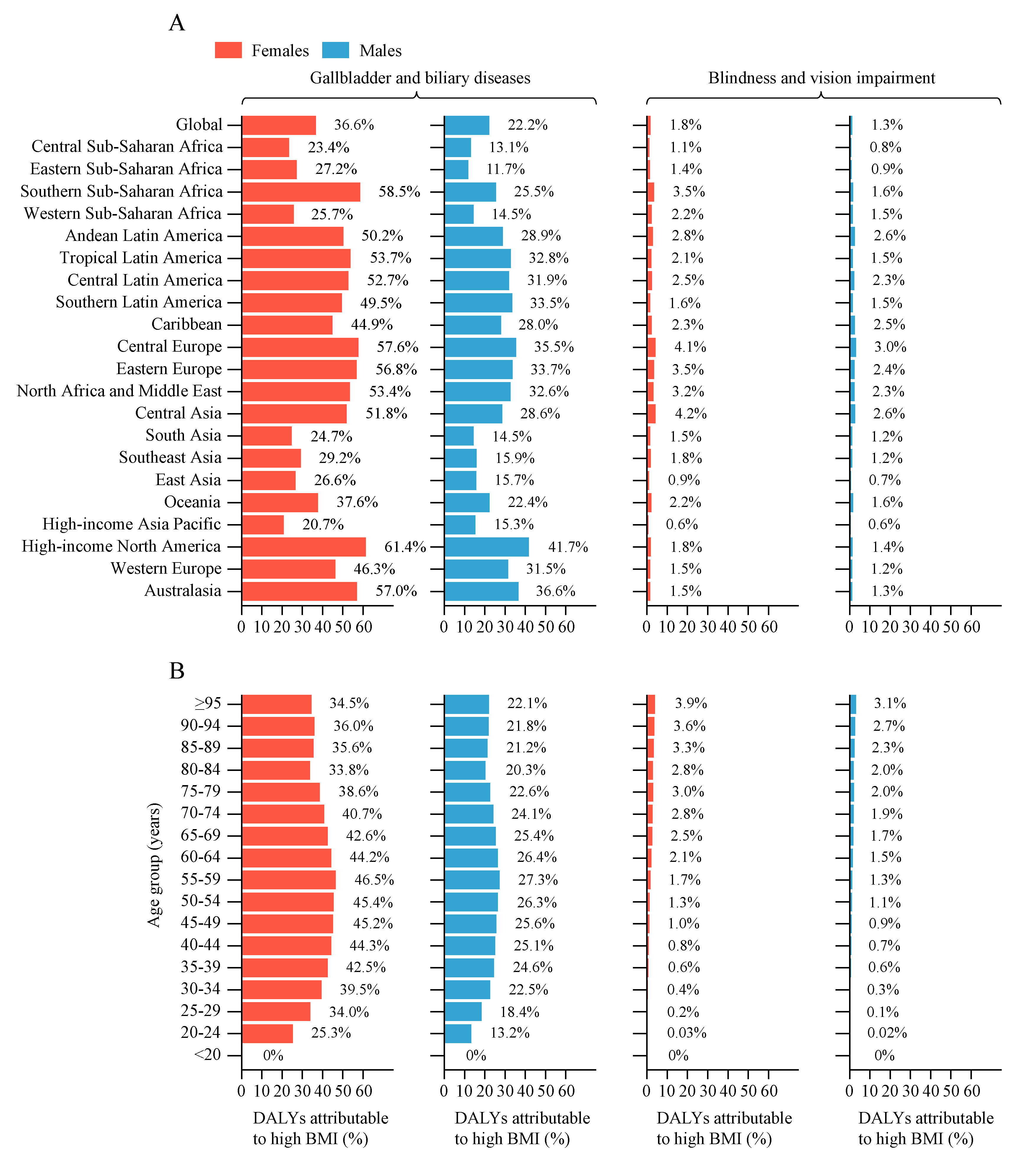

Supplement: S10 Fig — (A) By region. (B) By age group. BMI, body mass index; DALY, disability-adjusted life year. (TIF) [file pmed.1003198.s011.tif]

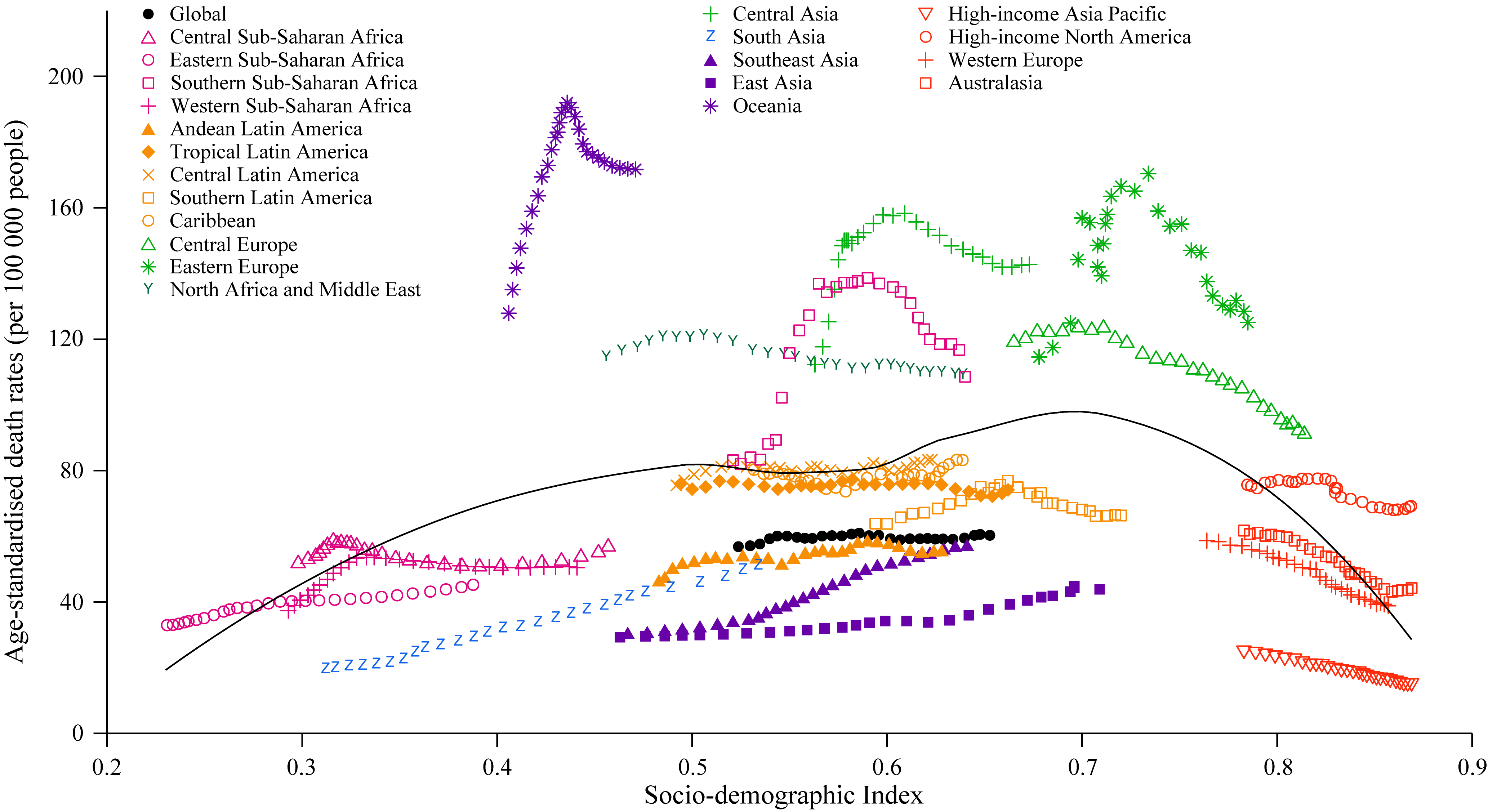

Supplement: S11 Fig — For each region, points from left to right depict estimates from each year from 1990 to 2017. DALY, disability-adjusted life year; GBD, Global Burden of Disease Study. (TIF) [file pmed.1003198.s012.tif]
