# Supplementary material for: The global burden of disease attributable to high body mass index in 195 countries and territories, 1990–2017: An analysis of the Global Burden of Disease Study
Source: PLoS Med. 2020 Jul 28;17(7):e1003198. doi: 10.1371/journal.pmed.1003198 (PMC7386577; doi:10.1371/journal.pmed.1003198)
Supplement: S1 Table — (DOCX) [file pmed.1003198.s013.docx]

**S1 Table. Age-standardised Deaths and DALYs attributable to high body mass index for both sexes combined in 2017 and percentage change from 1990 to 2017, by location.**

|  | **Deaths** |  |  |  | **DALYs** |  |  |  |
| --- | --- | --- | --- | --- | --- | --- | --- | --- |
|  | **2017 age-standardised**  **rates per 100 000 people** | **Percentage**  **change in age-standardised rates, 1990–2017** | **2017 age-standardised PAF** | **Percentage**  **change in age-standardised PAF, 1990–2017** | **2017 age-standardised**  **rates per 100 000 people** | **Percentage**  **change in age-standardised rates, 1990–2017** | **2017 age-standardised PAF** | **Percentage**  **change in age-standardised PAF, 1990–2017** |
| Global | 60.1  (37.9, 85.5) | 6.0%  (-4.5, 23.9) | 8.2%  (5.1, 11.6) | 55.1%  (39.7, 81.4)* | 1816.9  (1211.2, 2494.9) | 19.3%  (4.8, 43.2)* | 5.5%  (3.7, 7.5) | 76.8%  (55.8, 111.5)* |
| Low-SDI quintile | 38.8  (19.8, 63.1) | 56.3%  (22.7, 135.8)* | 3.4%  (1.8, 5.6) | 145.5%  (92.8, 269.8)* | 1240.2  (672.0, 1912.3) | 67.8%  (29.5, 152.2)* | 2.6%  (1.4, 4.0) | 189.2%  (123.9, 335.1)* |
| Low-middle-SDI quintile | 69.9  (44.6, 99.9) | 66.0%  (34.4, 126.0)* | 7.0%  (4.4, 10.0) | 130.6%  (87.3, 214.8)* | 2134.7  (1409.6, 2944.1) | 72.9%  (39.5, 132.8)* | 5.1%  (3.4, 7.0) | 157.2%  (108.2, 246.3)* |
| Middle-SDI quintile | 58.8  (36.2, 85.8) | 47.4%  (20.7, 99.3)* | 8.3%  (5.1, 12.1) | 113.1%  (74.7, 187.4)* | 1824.0  (1189.3, 2526.6) | 56.7%  (26.9, 111.3)* | 6.6%  (4.4, 9.1) | 136.5%  (92.6, 218.5)* |
| High-middle-SDI quintile | 75.8  (48.1, 107.4) | -4.0%  (-12.4, 9.7) | 11.7%  (7.4, 16.4) | 43.2%  (31.4, 63.9)* | 2128.5  (1418.2, 2902.1) | 2.2%  (-8.2, 18.7) | 8.6%  (5.8, 11.7) | 51.5%  (36.6, 75.0)* |
| High-SDI quintile | 44.9  (29.3, 62.2) | -26.9%  (-32.9, -16.0)* | 10.3%  (6.7, 14.3) | 11.7%  (2.5, 28.8)* | 1462.3  (1007.4, 1963.7) | -12.7%  (-21.5, 2.4) | 7.3%  (5.1, 9.6) | 12.1%  (2.1, 30.0)* |
| Central Sub-Saharan Africa | 56.7  (30.1, 90.3) | 9.7%  (-7.8, 43.7) | 4.2%  (2.2, 6.6) | 55.3%  (34.0, 100.7)* | 1771.3  (982.4, 2683.0) | 18.1%  (-0.9, 56.1) | 3.1%  (1.8, 4.7) | 80.6%  (54.0, 135.2)* |
| Angola | 59.4  (31.0, 93.6) | 85.6%  (14.7, 379.0)* | 4.9%  (2.6, 7.5) | 230.7%  (113.0, 737.7)* | 1929.6  (1108.4, 2850.6) | 105.3%  (26.4, 442.6)* | 3.8%  (2.2, 5.4) | 311.4%  (156.4, 964.4)* |
| Central African Republic | 64.8  (29.9, 108.7) | 50.3%  (12.7, 152.9)* | 3.0%  (1.4, 5.0) | 67.5%  (29.3, 176.9)* | 2030.5  (969.7, 3342.6) | 59.2%  (19.1, 165.3)* | 2.1%  (1.0, 3.4) | 71.5%  (27.9, 185.0)* |
| Congo | 102.1  (62.3, 147.9) | 13.8%  (-13.7, 72.1) | 7.5%  (4.7, 10.5) | 68.4%  (35.6, 143.9)* | 3065.6  (2012.4, 4250.3) | 16.9%  (-10.8, 75.4) | 5.7%  (3.9, 7.7) | 79.3%  (42.8, 164.8)* |
| Democratic Republic of the Congo | 50.2  (23.8, 83.7) | -6.2%  (-24.3, 22.9) | 3.7%  (1.8, 6.1) | 24.6%  (6.6, 53.4)* | 1559.7  (813.9, 2468.7) | 0.3%  (-17.4, 29.5) | 2.7%  (1.4, 4.3) | 43.9%  (22.4, 80.3)* |
| Equatorial Guinea | 82.4  (47.2, 128.5) | 75.0%  (-2.3, 371.8) | 7.6%  (4.9, 10.5) | 270.2%  (125.6, 850.2)* | 2571.0  (1648.4, 3703.3) | 88.1%  (6.6, 392.2)* | 5.5%  (3.9, 7.2) | 302.1%  (143.0, 964.2)* |
| Gabon | 95.6  (60.4, 134.0) | 28.7%  (0.0, 97.7)* | 9.3%  (6.1, 13.0) | 88.2%  (50.5, 182.4)* | 2952.4  (1995.6, 4033.5) | 32.1%  (4.3, 93.2)* | 7.0%  (4.8, 9.2) | 90.8%  (52.8, 176.9)* |
| Eastern Sub-Saharan Africa | 45.1  (23.4, 71.9) | 37.4%  (2.0, 132.3)* | 3.9%  (2.0, 6.3) | 138.2%  (77.3, 302.0)* | 1341.2  (752.8, 2028.4) | 43.3%  (5.1, 144.1)* | 2.8%  (1.6, 4.2) | 166.8%  (95.9, 353.6)* |
| Burundi | 28.9  (9.7, 57.2) | -30.5%  (-45.7, 6.2) | 2.1%  (0.7, 4.1) | 14.9%  (-3.2, 69.0) | 837.7  (300.0, 1574.9) | -29.9%  (-45.3, 2.7) | 1.5%  (0.5, 2.8) | 21.3%  (1.1, 76.7)* |
| Comoros | 52.4  (25.9, 85.7) | 1.8%  (-24.6, 68.5) | 5.3%  (2.7, 8.5) | 63.2%  (28.6, 162.9)* | 1605.4  (862.6, 2473.4) | 6.8%  (-20.0, 80.5) | 4.2%  (2.3, 6.4) | 87.6%  (43.6, 209.6)* |
| Djibouti | 71.3  (40.2, 110.3) | 135.0%  (40.1, 491.3)* | 7.0%  (4.3, 10.0) | 215.5%  (103.6, 670.8)* | 2077.1  (1257.2, 3043.3) | 140.8%  (46.5, 490.7)* | 5.3%  (3.5, 7.2) | 247.0%  (122.1, 731.9)* |
| Eritrea | 59.1  (29.5, 95.7) | 55.0%  (4.5, 209.8)* | 4.0%  (2.0, 6.3) | 272.0%  (161.0, 625.7)* | 1605.6  (839.6, 2480.6) | 49.3%  (1.8, 188.5)* | 2.8%  (1.5, 4.3) | 346.8%  (211.3, 740.1)* |
| Ethiopia | 29.5  (13.0, 50.6) | 9.5%  (-28.7, 192.2) | 3.1%  (1.3, 5.2) | 171.5%  (77.4, 621.1)* | 866.9  (413.4, 1403.2) | 14.7%  (-26.9, 194.0) | 2.1%  (1.0, 3.5) | 184.0%  (80.6, 630.6)* |
| Kenya | 50.6  (28.5, 77.1) | 69.5%  (31.3, 177.5)* | 4.5%  (2.6, 6.9) | 95.8%  (53.0, 219.9)* | 1550.6  (962.7, 2231.7) | 70.1%  (34.0, 166.1)* | 3.4%  (2.1, 4.9) | 108.0%  (65.2, 223.2)* |
| Madagascar | 56.7  (26.6, 94.8) | 33.0%  (0.9, 101.2)* | 4.4%  (2.1, 7.2) | 73.9%  (40.2, 155.0)* | 1686.8  (824.8, 2721.3) | 31.2%  (0.8, 97.9)* | 3.2%  (1.6, 5.2) | 91.7%  (51.7, 183.9)* |
| Malawi | 51.0  (24.2, 82.7) | 87.4%  (21.2, 368.7)* | 4.2%  (2.0, 6.8) | 217.5%  (106.6, 709.2)* | 1513.2  (770.2, 2358.0) | 86.7%  (22.7, 341.9)* | 2.9%  (1.5, 4.4) | 254.5%  (133.2, 722.0)* |
| Mozambique | 59.1  (27.7, 98.1) | 112.1%  (35.3, 499.8)* | 3.8%  (1.8, 6.3) | 169.8%  (75.7, 643.7)* | 1800.5  (893.0, 2848.5) | 125.0%  (42.7, 500.3)* | 2.7%  (1.4, 4.3) | 228.6%  (109.0, 769.9)* |
| Rwanda | 39.1  (18.0, 67.0) | -8.6%  (-34.3, 76.0) | 3.9%  (1.8, 6.6) | 111.4%  (55.5, 305.0)* | 1083.1  (545.5, 1719.0) | -9.1%  (-34.5, 71.8) | 2.7%  (1.4, 4.3) | 112.6%  (54.1, 300.2)* |
| Somalia | 50.8  (19.4, 95.4) | 32.7%  (-7.4, 168.6) | 3.2%  (1.3, 5.8) | 88.1%  (37.0, 275.8)* | 1500.5  (632.9, 2653.8) | 37.7%  (-6.3, 188.2) | 2.4%  (1.1, 4.0) | 124.6%  (58.2, 367.3)* |
| South Sudan | 42.4  (16.6, 78.0) | 29.0%  (-12.5, 155.0) | 2.8%  (1.2, 5.0) | 65.9%  (20.4, 214.5)* | 1260.8  (527.1, 2205.6) | 39.1%  (-5.7, 173.3) | 1.9%  (0.8, 3.1) | 81.1%  (26.9, 248.2)* |
| Tanzania | 54.1  (31.9, 81.3) | 32.2%  (0.3, 111.4)* | 5.3%  (3.1, 7.8) | 110.7%  (65.3, 236.7)* | 1604.2  (1024.6, 2242.9) | 43.6%  (8.9, 136.6)* | 3.6%  (2.3, 5.1) | 148.6%  (91.1, 302.1)* |
| Uganda | 37.7  (17.0, 63.2) | 64.5%  (12.0, 283.0)* | 3.5%  (1.6, 5.8) | 223.9%  (123.0, 629.4)* | 1149.6  (584.8, 1821.8) | 74.9%  (20.2, 288.0)* | 2.5%  (1.3, 3.8) | 291.4%  (167.3, 759.0)* |
| Zambia | 55.1  (28.7, 88.4) | 21.7%  (-8.0, 102.5) | 4.4%  (2.3, 6.9) | 96.0%  (52.0, 221.3)* | 1650.2  (934.9, 2484.2) | 22.9%  (-6.1, 101.7) | 3.2%  (1.8, 4.7) | 115.0%  (66.7, 251.0)* |
| Southern Sub-Saharan Africa | 108.6  (78.1, 140.1) | 30.6%  (20.1, 44.7)* | 9.8%  (7.1, 12.7) | 30.2%  (19.4, 44.3)* | 3075.2  (2305.1, 3858.1) | 19.8%  (11.9, 31.4)* | 6.4%  (4.8, 7.9) | 30.3%  (21.1, 43.1)* |
| Botswana | 113.7  (83.4, 146.5) | 63.8%  (22.2, 143.1)* | 11.6%  (8.6, 14.8) | 107.5%  (60.5, 198.7)* | 3123.4  (2409.6, 3839.1) | 55.9%  (20.4, 116.8)* | 7.9%  (6.2, 9.7) | 103.8%  (60.4, 181.2)* |
| Lesotho | 157.9  (98.8, 229.1) | 130.1%  (67.1, 245.5)* | 8.5%  (5.6, 11.7) | 74.9%  (37.9, 154.0)* | 4374.6  (2927.9, 6017.6) | 122.0%  (68.3, 223.2)* | 5.8%  (3.9, 7.8) | 75.7%  (39.6, 147.3)* |
| Namibia | 82.0  (54.3, 112.5) | -6.4%  (-25.7, 28.8) | 7.8%  (5.3, 10.4) | 23.8%  (2.8, 64.1)* | 2368.1  (1687.3, 3071.1) | -5.1%  (-21.3, 24.0) | 5.2%  (3.8, 6.8) | 19.1%  (0.3, 53.0)* |
| South Africa | 108.8  (78.7, 138.8) | 23.5%  (14.3, 36.1)* | 10.5%  (7.6, 13.5) | 22.6%  (12.7, 34.9)* | 3079.6  (2316.5, 3852.2) | 10.7%  (3.4, 20.2)* | 6.8%  (5.2, 8.4) | 24.6%  (15.8, 35.5)* |
| Swaziland | 209.1  (139.6, 286.8) | 39.5%  (9.7, 82.9)* | 13.9%  (9.8, 17.8) | 30.3%  (12.5, 57.1)* | 5703.9  (3980.4, 7463.9) | 40.6%  (14.5, 77.1)* | 9.3%  (6.7, 11.6) | 27.1%  (9.7, 51.4)* |
| Zimbabwe | 87.4  (55.6, 126.7) | 60.3%  (28.6, 109.0)* | 6.2%  (4.1, 8.8) | 54.0%  (25.0, 101.4)* | 2676.9  (1791.8, 3706.3) | 64.7%  (36.1, 103.9)* | 4.8%  (3.3, 6.4) | 69.6%  (39.1, 118.0)* |
| Western Sub-Saharan Africa | 50.5  (29.5, 76.2) | 35.1%  (10.3, 80.8)* | 4.3%  (2.5, 6.4) | 88.4%  (60.7, 149.5)* | 1536.8  (942.0, 2214.4) | 38.7%  (16.1, 82.7)* | 2.9%  (1.8, 4.0) | 112.6%  (79.7, 177.6)* |
| Benin | 65.4  (39.0, 98.1) | 72.3%  (24.4, 193.0)* | 5.7%  (3.5, 8.2) | 134.5%  (79.0, 288.4)* | 2146.6  (1387.7, 3037.3) | 80.4%  (33.4, 204.8)* | 4.3%  (2.8, 5.9) | 172.0%  (106.7, 357.6)* |
| Burkina Faso | 57.0  (29.2, 91.9) | 107.5%  (52.2, 279.5)* | 4.4%  (2.2, 6.9) | 195.7%  (117.7, 435.0)* | 1652.8  (917.2, 2537.9) | 99.9%  (48.5, 269.4)* | 2.9%  (1.6, 4.3) | 217.3%  (135.3, 486.0)* |
| Cameroon | 90.8  (54.5, 132.2) | 17.6%  (-4.4, 49.4) | 7.3%  (4.6, 10.1) | 47.1%  (27.1, 78.5)* | 2564.9  (1646.0, 3541.5) | 19.2%  (-0.5, 48.2) | 4.9%  (3.3, 6.7) | 63.8%  (40.6, 96.0)* |
| Cape Verde | 49.0  (29.9, 72.1) | 65.9%  (30.4, 159.2)* | 7.7%  (4.7, 11.1) | 109.1%  (67.2, 225.9)* | 1626.3  (1058.7, 2294.0) | 66.4%  (31.0, 156.2)* | 5.8%  (3.8, 7.9) | 135.4%  (88.0, 264.4)* |
| Chad | 37.9  (17.7, 63.9) | 88.1%  (38.9, 224.1)* | 2.7%  (1.3, 4.5) | 131.7%  (74.1, 292.0)* | 1193.2  (579.6, 1927.2) | 95.6%  (45.4, 233.8)* | 1.9%  (0.9, 3.0) | 169.6%  (99.3, 362.2)* |
| Cote d'Ivoire | 69.7  (39.1, 105.7) | 22.1%  (-1.1, 61.2) | 5.5%  (3.1, 8.2) | 59.0%  (35.8, 104.4)* | 2188.6  (1337.0, 3161.0) | 27.1%  (4.7, 65.3)* | 4.0%  (2.5, 5.7) | 79.1%  (48.3, 131.9)* |
| The Gambia | 81.5  (46.9, 124.1) | 77.1%  (33.6, 174.4)* | 6.9%  (4.1, 10.3) | 113.4%  (72.2, 213.8)* | 2459.5  (1513.9, 3567.7) | 79.0%  (35.8, 177.4)* | 5.2%  (3.3, 7.5) | 140.9%  (90.3, 265.6)* |
| Ghana | 84.0  (53.6, 120.4) | 104.6%  (53.8, 217.6)* | 7.3%  (4.7, 10.3) | 153.9%  (100.2, 290.2)* | 2479.1  (1665.8, 3431.4) | 91.0%  (47.1, 180.4)* | 5.4%  (3.7, 7.3) | 166.0%  (112.1, 293.5)* |
| Guinea | 63.3  (32.3, 100.3) | 80.2%  (40.8, 166.3)* | 4.4%  (2.3, 6.9) | 124.3%  (79.2, 229.0)* | 1901.8  (1025.2, 2871.5) | 84.4%  (45.3, 174.2)* | 3.2%  (1.8, 4.8) | 182.0%  (121.8, 318.2)* |
| Guinea-Bissau | 72.4  (35.7, 117.0) | 36.2%  (3.8, 116.2)* | 4.7%  (2.4, 7.4) | 97.8%  (58.9, 194.8)* | 2218.5  (1177.2, 3436.5) | 40.1%  (8.6, 124.2)* | 3.6%  (1.9, 5.5) | 127.2%  (79.5, 250.8)* |
| Liberia | 70.6  (42.5, 103.9) | 60.8%  (19.2, 167.6)* | 6.0%  (3.7, 8.6) | 183.6%  (122.0, 359.4)* | 2355.8  (1533.7, 3307.0) | 71.7%  (29.1, 186.9)* | 4.6%  (3.0, 6.3) | 280.1%  (189.7, 529.5)* |
| Mali | 44.9  (21.7, 73.9) | 44.4%  (5.3, 153.8)* | 3.6%  (1.8, 5.8) | 134.9%  (77.3, 314.3)* | 1387.3  (726.1, 2172.3) | 56.7%  (13.0, 188.8)* | 2.3%  (1.2, 3.6) | 169.2%  (95.2, 393.7)* |
| Mauritania | 82.1  (52.8, 114.7) | -6.6%  (-25.0, 24.1) | 9.2%  (6.1, 12.6) | 56.8%  (31.8, 103.2)* | 2346.8  (1604.4, 3171.0) | -5.0%  (-21.4, 22.5) | 6.5%  (4.5, 8.5) | 66.1%  (40.1, 110.1)* |
| Niger | 35.4  (16.1, 60.0) | 18.4%  (-6.7, 76.7) | 2.9%  (1.4, 4.9) | 96.3%  (60.0, 189.4)* | 1114.5  (545.6, 1813.6) | 25.3%  (-0.1, 84.6) | 2.0%  (1.0, 3.3) | 149.3%  (99.0, 263.3)* |
| Nigeria | 33.5  (16.5, 57.8) | 5.0%  (-26.0, 60.0) | 3.1%  (1.6, 4.9) | 51.9%  (21.2, 109.7)* | 1038.6  (560.9, 1650.7) | 11.6%  (-15.9, 60.3) | 1.9%  (1.1, 2.9) | 72.9%  (36.2, 139.8)* |
| Sao Tome and Principe | 71.5  (42.5, 106.3) | 80.8%  (36.2, 188.4)* | 7.3%  (4.4, 10.9) | 106.8%  (61.8, 219.8)* | 2202.4  (1411.6, 3099.1) | 77.0%  (35.3, 179.6)* | 6.0%  (3.9, 8.4) | 155.8%  (99.8, 293.5)* |
| Senegal | 64.4  (37.6, 96.0) | 24.3%  (2.6, 61.9)* | 6.2%  (3.7, 9.1) | 80.1%  (54.2, 129.8)* | 1957.0  (1225.4, 2779.6) | 24.6%  (5.4, 56.9)* | 4.7%  (3.0, 6.7) | 107.7%  (79.0, 158.8)* |
| Sierra Leone | 62.7  (32.5, 98.9) | 87.1%  (32.5, 265.5)* | 4.5%  (2.3, 7.0) | 151.9%  (82.7, 382.7)* | 1923.9  (1078.8, 2891.0) | 91.5%  (35.9, 276.2)* | 3.1%  (1.8, 4.7) | 202.4%  (116.9, 488.7)* |
| Togo | 56.5  (30.6, 88.4) | 49.2%  (15.3, 126.4)* | 4.8%  (2.7, 7.4) | 91.4%  (56.3, 177.0)* | 1712.4  (1006.3, 2547.2) | 53.4%  (21.1, 124.4)* | 3.5%  (2.1, 5.1) | 116.4%  (74.7, 214.7)* |
| Andean Latin America | 55.1  (34.3, 77.7) | 20.0%  (-0.7, 65.7) | 10.3%  (6.4, 14.4) | 97.5%  (65.9, 170.1)* | 1759.5  (1190.3, 2378.1) | 19.8%  (1.0, 59.7)* | 7.3%  (4.9, 9.7) | 110.2%  (78.1, 178.2)* |
| Bolivia | 77.6  (45.4, 117.6) | 34.6%  (-2.0, 137.6) | 9.8%  (5.8, 14.1) | 129.2%  (73.8, 295.7)* | 2272.3  (1420.2, 3279.1) | 29.6%  (-4.6, 121.6) | 7.4%  (4.7, 10.4) | 150.1%  (88.1, 323.2)* |
| Ecuador | 77.8  (50.6, 106.9) | 25.8%  (8.4, 54.8)* | 12.9%  (8.5, 17.4) | 60.9%  (42.4, 96.5)* | 2369.8  (1636.3, 3073.7) | 13.5%  (1.3, 32.8)* | 9.3%  (6.6, 11.9) | 52.2%  (36.6, 76.4)* |
| Peru | 38.6  (22.9, 56.6) | 8.1%  (-15.6, 64.6) | 8.7%  (5.3, 12.6) | 96.9%  (61.6, 190.1)* | 1311.8  (845.6, 1844.7) | 18.0%  (-6.1, 73.1) | 6.1%  (3.9, 8.3) | 124.6%  (83.1, 229.6)* |
| Tropical Latin America | 74.1  (52.7, 97.3) | -2.4%  (-17.9, 30.2) | 11.9%  (8.4, 15.6) | 52.2%  (28.2, 102.9)* | 2188.0  (1607.2, 2807.1) | -3.4%  (-17.8, 25.5) | 7.9%  (5.8, 10.0) | 48.8%  (27.2, 93.1)* |
| Brazil | 73.9  (52.7, 97.0) | -3.2%  (-18.6, 29.5) | 11.8%  (8.4, 15.5) | 51.9%  (27.7, 103.1)* | 2180.8  (1605.2, 2793.1) | -4.2%  (-18.4, 24.7) | 7.8%  (5.8, 9.9) | 48.3%  (26.6, 92.9)* |
| Paraguay | 83.0  (53.2, 118.9) | 39.9%  (11.3, 90.6)* | 13.4%  (8.7, 18.5) | 60.4%  (37.8, 109.6)* | 2503.8  (1683.9, 3429.0) | 37.9%  (14.5, 74.9)* | 9.7%  (6.6, 12.9) | 63.7%  (41.7, 104.0)* |
| Central Latin America | 83.3  (55.7, 111.1) | 10.3%  (0.5, 26.1)* | 14.1%  (9.4, 18.9) | 54.3%  (40.8, 76.1)* | 2603.3  (1806.9, 3395.7) | 12.7%  (4.1, 26.8)* | 10.2%  (7.2, 13.0) | 54.6%  (42.4, 74.2)* |
| Colombia | 45.8  (29.1, 65.9) | -33.6%  (-43.5, -18.9)* | 10.4%  (6.6, 14.7) | 19.9%  (6.0, 45.1)* | 1407.7  (928.0, 1942.5) | -26.6%  (-35.6, -11.6)* | 6.6%  (4.4, 8.9) | 17.5%  (4.0, 41.1)* |
| Costa Rica | 56.7  (35.4, 79.6) | 0.7%  (-11.9, 24.3) | 11.8%  (7.4, 16.4) | 29.9%  (15.2, 59.7)* | 1719.8  (1141.9, 2319.7) | 5.4%  (-5.7, 24.2) | 8.3%  (5.5, 10.9) | 23.8%  (12.1, 45.6)* |
| El Salvador | 94.7  (58.9, 135.4) | 57.0%  (23.2, 112.5)* | 13.4%  (8.6, 18.6) | 92.5%  (56.5, 153.2)* | 2835.2  (1895.6, 3901.0) | 43.4%  (17.2, 83.3)* | 9.7%  (6.4, 13.0) | 99.1%  (66.6, 150.5)* |
| Guatemala | 69.7  (42.7, 99.5) | 84.2%  (38.7, 216.3)* | 9.2%  (5.7, 13.0) | 207.0%  (134.5, 420.7)* | 2303.7  (1526.3, 3196.9) | 88.2%  (44.3, 208.7)* | 7.1%  (4.8, 9.6) | 214.7%  (141.9, 412.3)* |
| Honduras | 77.3  (46.1, 113.9) | 53.9%  (15.8, 130.7)* | 10.8%  (6.6, 15.5) | 85.0%  (52.9, 163.6)* | 2310.4  (1464.1, 3269.7) | 42.7%  (10.5, 117.2)* | 8.1%  (5.3, 11.1) | 103.7%  (65.7, 198.6)* |
| Mexico | 103.0  (69.1, 134.7) | 23.3%  (13.5, 38.4)* | 16.2%  (10.8, 21.2) | 58.4%  (46.0, 78.3)* | 3236.4  (2287.3, 4146.8) | 22.3%  (14.5, 34.2)* | 12.4%  (8.8, 15.5) | 58.2%  (47.8, 73.8)* |
| Nicaragua | 69.2  (43.7, 96.5) | 22.6%  (-1.2, 65.4) | 14.3%  (9.2, 19.8) | 85.8%  (56.7, 147.9)* | 2160.6  (1465.7, 2908.2) | 20.9%  (0.8, 57.9)* | 9.5%  (6.4, 12.8) | 94.6%  (65.2, 150.8)* |
| Panama | 52.2  (32.7, 73.5) | 71.9%  (23.3, 238.9)* | 11.0%  (6.9, 15.4) | 126.9%  (63.0, 353.3)* | 1658.1  (1107.2, 2226.1) | 79.6%  (31.2, 231.2)* | 7.3%  (5.0, 9.8) | 113.1%  (55.2, 293.0)* |
| Venezuela | 92.6  (59.5, 129.4) | 4.2%  (-12.3, 29.5) | 14.6%  (9.6, 19.8) | 30.6%  (17.1, 56.0)* | 2692.8  (1833.9, 3628.1) | 3.2%  (-10.4, 23.4) | 9.8%  (6.8, 12.8) | 22.5%  (10.3, 42.3)* |
| Southern Latin America | 66.3  (41.4, 92.2) | 3.9%  (-15.3, 47.5) | 11.4%  (7.1, 15.8) | 43.9%  (20.0, 104.2)* | 1978.0  (1308.9, 2685.1) | 10.3%  (-9.5, 54.0) | 8.3%  (5.5, 11.0) | 44.7%  (20.8, 102.5)* |
| Argentina | 69.9  (43.3, 98.1) | 9.1%  (-13.6, 63.5) | 11.3%  (6.9, 15.7) | 43.6%  (17.8, 113.0)* | 2056.6  (1370.0, 2776.6) | 14.2%  (-8.9, 66.3) | 8.4%  (5.6, 11.1) | 46.6%  (20.0, 112.5)* |
| Chile | 59.2  (35.8, 83.9) | -9.0%  (-24.1, 20.6) | 12.0%  (7.3, 16.7) | 46.0%  (24.8, 91.5)* | 1815.9  (1160.0, 2496.9) | 1.2%  (-13.9, 31.8) | 8.4%  (5.4, 11.2) | 42.2%  (22.9, 85.4)* |
| Uruguay | 62.6  (39.3, 87.1) | 2.8%  (-16.7, 47.0) | 10.5%  (6.6, 14.4) | 36.0%  (12.7, 90.1)* | 1938.7  (1291.3, 2628.9) | 7.8%  (-10.9, 48.4) | 8.1%  (5.4, 10.6) | 35.1%  (13.3, 85.3)* |
| Caribbean | 83.1  (54.9, 112.2) | 3.6%  (-6.4, 20.3) | 11.4%  (7.5, 15.4) | 32.5%  (21.0, 52.4)* | 2601.0  (1826.7, 3403.7) | 7.9%  (-2.2, 24.0) | 8.0%  (5.6, 10.4) | 37.2%  (24.2, 57.8)* |
| Antigua and Barbuda | 81.1  (52.2, 112.3) | -4.2%  (-15.3, 15.5) | 13.8%  (8.9, 19.1) | 16.9%  (4.7, 40.3)* | 2538.3  (1720.7, 3376.9) | -4.5%  (-14.3, 12.0) | 10.4%  (7.1, 13.5) | 10.8%  (0.4, 29.7)* |
| The Bahamas | 108.7  (70.5, 146.6) | -8.9%  (-17.8, 1.9) | 15.1%  (9.9, 20.2) | 6.5%  (-0.7, 17.0) | 3308.2  (2301.4, 4268.4) | -7.2%  (-14.7, 1.9) | 11.4%  (7.9, 14.5) | 6.7%  (-0.3, 16.4) |
| Barbados | 82.7  (54.1, 112.3) | -15.2%  (-24.8, 0.1) | 13.7%  (9.1, 18.5) | 6.8%  (-3.1, 25.7) | 2588.8  (1794.3, 3380.9) | -7.7%  (-17.0, 6.9) | 10.9%  (7.7, 14.0) | 8.9%  (-1.6, 25.9) |
| Belize | 101.0  (68.6, 130.8) | 22.2%  (3.9, 57.0)* | 14.9%  (10.1, 19.3) | 43.3%  (22.9, 83.4)* | 3255.1  (2380.0, 4090.6) | 27.8%  (12.4, 56.6)* | 10.9%  (8.0, 13.5) | 43.0%  (25.0, 75.8)* |
| Bermuda | 58.6  (36.6, 79.4) | -52.7%  (-57.4, -47.9)* | 13.8%  (8.8, 18.6) | -15.1%  (-22.4, -9.0)* | 1872.3  (1314.8, 2449.5) | -45.3%  (-49.9, -40.5)* | 10.3%  (7.2, 12.9) | -18.1%  (-22.9, -12.9)* |
| Cuba | 57.8  (35.1, 82.9) | -12.8%  (-27.7, 5.2) | 10.7%  (6.6, 15.1) | 14.8%  (-1.4, 36.7) | 1814.9  (1191.6, 2504.9) | -10.1%  (-25.5, 6.7) | 8.6%  (5.7, 11.6) | 13.8%  (-5.1, 32.8) |
| Dominica | 90.7  (56.0, 127.3) | 10.6%  (-3.0, 36.9) | 11.7%  (7.3, 16.4) | 12.0%  (-0.6, 37.6) | 2722.9  (1809.4, 3666.6) | 12.1%  (0.7, 31.7)* | 8.5%  (5.7, 11.3) | 4.4%  (-8.0, 24.4) |
| Dominican Republic | 98.2  (61.0, 137.4) | 74.7%  (39.0, 140.6)* | 13.7%  (8.7, 18.9) | 82.2%  (52.6, 144.5)* | 3050.8  (2080.7, 4072.6) | 68.1%  (37.5, 123.1)* | 9.9%  (6.7, 13.0) | 100.5%  (65.8, 163.1)* |
| Grenada | 92.1  (58.9, 129.5) | -5.3%  (-17.2, 20.6) | 12.8%  (8.2, 17.7) | 32.7%  (16.0, 69.6)* | 2944.6  (2001.2, 3976.0) | 0.5%  (-12.1, 25.7) | 10.7%  (7.3, 14.4) | 29.9%  (13.7, 63.1)* |
| Guyana | 134.6  (83.1, 190.2) | 4.7%  (-10.6, 32.1) | 14.3%  (8.9, 19.9) | 26.9%  (12.2, 56.2)* | 4118.6  (2683.5, 5619.8) | 6.7%  (-7.7, 30.8) | 11.1%  (7.3, 14.7) | 28.7%  (13.3, 59.2)* |
| Haiti | 89.6  (49.7, 136.9) | 5.7%  (-19.5, 60.9) | 7.3%  (4.2, 10.9) | 53.3%  (26.7, 123.5)* | 2722.8  (1604.8, 4018.9) | 5.4%  (-17.4, 58.8) | 5.6%  (3.4, 8.0) | 67.3%  (35.2, 145.6)* |
| Jamaica | 104.2  (66.2, 144.8) | 36.2%  (11.3, 76.8)* | 15.1%  (9.9, 20.7) | 35.1%  (16.0, 71.5)* | 3148.7  (2119.0, 4178.6) | 42.8%  (20.3, 77.0)* | 11.2%  (7.8, 14.7) | 39.1%  (20.6, 70.3)* |
| Puerto Rico | 82.8  (59.4, 104.5) | -18.4%  (-25.8, -6.5)* | 16.0%  (11.6, 19.9) | 12.7%  (3.3, 28.8)* | 2827.4  (2155.1, 3495.6) | -7.3%  (-15.6, 5.5) | 11.7%  (9.2, 13.8) | 8.8%  (0.2, 22.8)* |
| Saint Lucia | 86.3  (55.0, 118.0) | -15.5%  (-26.1, 4.3) | 13.0%  (8.3, 17.9) | 21.8%  (7.5, 49.2)* | 2761.6  (1920.1, 3650.1) | -10.5%  (-20.2, 8.5) | 10.4%  (7.3, 13.5) | 15.1%  (1.4, 38.7)* |
| Saint Vincent and the Grenadines | 107.5  (67.5, 149.1) | 24.6%  (5.3, 67.5)* | 14.1%  (8.9, 19.6) | 46.5%  (24.3, 95.2)* | 3490.3  (2338.1, 4633.3) | 32.4%  (13.8, 70.0)* | 11.3%  (7.6, 14.9) | 44.5%  (24.0, 87.1)* |
| Suriname | 114.2  (74.4, 155.6) | 14.6%  (-3.3, 44.3) | 14.4%  (9.5, 19.4) | 38.7%  (21.5, 71.7)* | 3598.9  (2484.6, 4693.6) | 20.3%  (3.2, 48.9)* | 10.9%  (7.6, 14.1) | 47.5%  (28.9, 80.9)* |
| Trinidad and Tobago | 125.9  (82.5, 175.1) | -19.2%  (-33.2, -0.5)* | 18.2%  (12.3, 24.1) | 13.0%  (1.4, 29.6)* | 3979.0  (2836.6, 5307.6) | -15.1%  (-27.5, 0.9) | 13.8%  (10.0, 17.5) | 4.2%  (-6.1, 18.7) |
| Virgin Islands, U.S. | 135.2  (91.3, 176.1) | -1.4%  (-15.9, 13.4) | 17.8%  (12.3, 22.9) | 8.3%  (1.2, 19.1)* | 3732.6  (2687.4, 4740.8) | 1.6%  (-10.7, 14.2) | 13.7%  (10.0, 16.9) | 11.4%  (3.3, 21.5)* |
| Central Europe | 91.1  (60.9, 123.3) | -23.5%  (-29.2, -14.0)* | 14.5%  (9.7, 19.6) | 16.7%  (7.9, 31.5)* | 2490.7  (1750.3, 3261.0) | -20.1%  (-26.4, -9.7)* | 10.4%  (7.3, 13.5) | 14.6%  (6.3, 28.8)* |
| Albania | 64.3  (35.5, 99.8) | 12.1%  (-9.8, 46.9) | 11.4%  (6.5, 16.9) | 41.4%  (24.1, 74.4)* | 1775.0  (1066.4, 2600.6) | 16.5%  (-2.9, 50.9) | 7.7%  (4.8, 10.9) | 59.5%  (36.8, 101.3)* |
| Bosnia and Herzegovina | 86.8  (53.9, 122.4) | 20.4%  (2.6, 55.0)* | 12.8%  (8.0, 18.0) | 40.7%  (20.5, 80.3)* | 2383.1  (1578.5, 3256.9) | 17.1%  (1.2, 45.6)* | 9.8%  (6.5, 13.3) | 42.0%  (23.8, 74.8)* |
| Bulgaria | 125.1  (78.7, 173.9) | -8.6%  (-17.5, 2.4) | 16.9%  (10.6, 23.4) | 18.3%  (8.4, 31.4)* | 3385.4  (2328.4, 4478.8) | -3.6%  (-10.8, 6.6) | 12.8%  (8.6, 16.9) | 18.3%  (10.4, 29.4)* |
| Croatia | 79.2  (49.9, 110.0) | -23.9%  (-31.8, -10.0)* | 13.6%  (8.6, 18.9) | 17.6%  (6.7, 37.3)* | 2202.9  (1484.3, 2974.2) | -19.8%  (-27.5, -6.5)* | 10.2%  (6.9, 13.4) | 13.3%  (4.3, 30.9)* |
| Czech Republic | 74.9  (47.6, 105.0) | -42.2%  (-48.5, -32.7)* | 14.0%  (8.9, 19.3) | 7.6%  (-2.7, 23.8) | 2093.0  (1401.3, 2826.7) | -36.6%  (-43.3, -26.7)* | 9.8%  (6.6, 12.9) | -3.1%  (-11.3, 10.8) |
| Hungary | 94.8  (61.9, 127.9) | -34.7%  (-40.8, -28.3)* | 15.0%  (9.9, 20.4) | 9.2%  (0.0, 18.9) | 2680.7  (1887.7, 3492.9) | -32.2%  (-37.2, -25.7)* | 11.1%  (7.8, 14.2) | 4.2%  (-1.7, 12.7) |
| Macedonia | 96.9  (60.3, 135.0) | -15.9%  (-23.5, -3.6)* | 16.1%  (10.1, 22.5) | 24.4%  (14.6, 41.5)* | 2789.4  (1812.4, 3786.9) | -5.0%  (-13.4, 8.8) | 11.6%  (7.5, 15.7) | 32.0%  (21.2, 50.9)* |
| Montenegro | 99.0  (61.3, 140.3) | -2.0%  (-11.6, 9.8) | 14.4%  (9.0, 20.3) | 8.9%  (1.1, 19.4)* | 2721.9  (1822.4, 3679.0) | -4.2%  (-11.7, 5.4) | 11.4%  (7.5, 15.2) | 12.5%  (6.3, 21.6)* |
| Poland | 77.2  (49.5, 105.3) | -31.6%  (-40.0, -18.2)* | 13.6%  (8.8, 18.5) | 12.1%  (-1.3, 33.5) | 2215.5  (1524.4, 2918.7) | -26.4%  (-35.2, -12.4)* | 9.6%  (6.6, 12.5) | 7.9%  (-4.0, 28.0) |
| Romania | 109.1  (78.6, 143.1) | -8.4%  (-18.9, 10.8) | 15.2%  (10.9, 20.0) | 31.7%  (17.4, 59.9)* | 2872.8  (2159.5, 3639.8) | -3.8%  (-13.9, 13.7) | 10.7%  (8.1, 13.6) | 34.1%  (21.2, 58.3)* |
| Serbia | 119.0  (73.7, 165.7) | 0.7%  (-10.9, 19.5) | 14.5%  (9.1, 20.2) | 18.0%  (4.9, 40.2)* | 2908.7  (1925.1, 3865.0) | -8.2%  (-17.2, 7.0) | 11.4%  (7.6, 15.0) | 26.4%  (14.8, 47.3)* |
| Slovakia | 94.0  (61.1, 129.7) | -31.8%  (-38.2, -22.8)* | 15.2%  (9.9, 21.0) | 7.4%  (-1.8, 20.6) | 2411.3  (1620.2, 3207.8) | -32.1%  (-37.9, -24.9)* | 10.3%  (6.9, 13.6) | -2.4%  (-9.6, 7.1) |
| Slovenia | 53.1  (33.9, 73.2) | -34.6%  (-42.9, -23.3)* | 11.9%  (7.6, 16.4) | 18.0%  (5.2, 36.2)* | 1610.7  (1099.8, 2171.3) | -25.2%  (-33.3, -13.6)* | 8.2%  (5.6, 10.6) | 10.2%  (0.4, 26.6)* |
| Eastern Europe | 125.1  (82.7, 170.7) | 9.2%  (-1.6, 25.2) | 14.7%  (9.8, 20.1) | 27.1%  (14.4, 45.8)* | 3271.6  (2286.1, 4320.7) | 9.9%  (0.5, 23.5)* | 10.1%  (7.0, 13.3) | 24.9%  (14.0, 40.7)* |
| Belarus | 122.5  (78.2, 169.1) | 20.2%  (7.2, 41.1)* | 15.9%  (10.0, 22.0) | 38.3%  (24.7, 61.5)* | 3125.2  (2115.0, 4200.1) | 13.6%  (3.6, 29.9)* | 11.1%  (7.4, 14.8) | 37.9%  (25.7, 56.8)* |
| Estonia | 93.6  (60.3, 130.0) | -21.5%  (-36.4, 1.3) | 16.7%  (10.7, 22.7) | 38.5%  (12.3, 78.3)* | 2413.8  (1713.1, 3158.2) | -25.4%  (-35.6, -11.3)* | 10.4%  (7.5, 13.3) | 15.8%  (2.0, 35.9)* |
| Latvia | 106.5  (67.4, 148.2) | -14.7%  (-25.8, 0.9) | 15.3%  (9.9, 21.1) | 21.9%  (10.7, 41.1)* | 2902.9  (2027.8, 3863.5) | -11.5%  (-21.5, 2.4) | 10.7%  (7.5, 14.1) | 19.4%  (9.5, 35.7)* |
| Lithuania | 98.3  (62.4, 135.7) | -2.0%  (-12.8, 17.1) | 14.0%  (8.8, 19.3) | 23.9%  (11.3, 46.8)* | 2613.4  (1737.0, 3507.4) | -1.5%  (-10.3, 13.5) | 9.5%  (6.3, 12.7) | 20.6%  (10.8, 38.5)* |
| Moldova | 115.1  (73.9, 159.8) | 14.4%  (-1.3, 47.1) | 14.8%  (9.5, 20.6) | 60.3%  (38.7, 105.4)* | 3188.6  (2176.0, 4284.7) | 20.8%  (6.2, 49.7)* | 10.3%  (6.9, 13.7) | 61.1%  (40.6, 100.7)* |
| Russian Federation | 123.3  (82.7, 167.3) | 5.5%  (-6.6, 23.2) | 14.8%  (9.9, 20.0) | 26.6%  (12.2, 47.7)* | 3224.7  (2252.1, 4249.1) | 6.6%  (-4.4, 22.2) | 10.0%  (7.0, 13.2) | 23.6%  (10.6, 41.9)* |
| Ukraine | 135.6  (88.4, 187.6) | 21.1%  (9.9, 36.8)* | 14.4%  (9.4, 19.9) | 24.4%  (13.4, 40.0)* | 3543.4  (2437.0, 4716.2) | 21.6%  (12.3, 34.1)* | 10.1%  (7.1, 13.5) | 24.5%  (15.5, 37.0)* |
| North Africa and Middle East | 109.5  (74.1, 147.8) | -4.7%  (-13.8, 9.1) | 15.7%  (10.7, 21.1) | 46.5%  (33.7, 67.6)* | 3256.0  (2318.9, 4252.5) | -2.2%  (-11.6, 11.3) | 10.4%  (7.4, 13.4) | 52.2%  (38.9, 72.5)* |
| Afghanistan | 177.1  (113.8, 247.9) | 66.6%  (17.0, 209.1)* | 13.0%  (8.4, 17.8) | 161.9%  (90.4, 374.2)* | 5241.4  (3449.9, 7237.2) | 61.9%  (14.6, 204.0)* | 9.2%  (6.1, 12.5) | 196.3%  (115.1, 441.3)* |
| Algeria | 96.0  (63.3, 130.3) | 4.7%  (-11.5, 33.5) | 17.6%  (11.6, 23.9) | 57.0%  (35.0, 100.7)* | 2796.6  (1958.5, 3648.9) | 3.1%  (-11.0, 27.3) | 11.5%  (8.1, 14.7) | 52.7%  (33.4, 85.7)* |
| Bahrain | 117.1  (80.6, 150.2) | -37.5%  (-45.0, -26.9)* | 23.8%  (16.8, 30.1) | 34.6%  (21.9, 55.0)* | 3301.3  (2409.8, 4170.9) | -32.6%  (-40.5, -23.2)* | 15.6%  (11.5, 19.1) | 12.9%  (3.5, 27.6)* |
| Egypt | 187.3  (120.0, 256.6) | 21.3%  (3.9, 44.4)* | 18.8%  (12.2, 25.5) | 55.0%  (38.2, 82.9)* | 5322.5  (3600.1, 7037.4) | 20.1%  (5.0, 39.3)* | 15.1%  (10.2, 19.6) | 84.0%  (63.4, 113.3)* |
| Iran | 83.4  (57.2, 113.3) | 0.7%  (-10.4, 21.6) | 13.8%  (9.5, 18.8) | 56.1%  (38.8, 88.0)* | 2315.3  (1661.1, 3034.6) | -0.5%  (-10.4, 16.3) | 8.8%  (6.4, 11.4) | 64.9%  (49.6, 92.1)* |
| Iraq | 84.1  (53.2, 114.0) | -60.0%  (-65.3, -53.3)* | 15.6%  (9.9, 20.9) | -13.1%  (-20.6, -3.3)* | 2792.7  (1903.3, 3667.1) | -53.0%  (-59.4, -45.6)* | 9.3%  (6.4, 12.0) | -24.8%  (-32.3, -15.9)* |
| Jordan | 106.7  (70.5, 143.6) | -23.6%  (-35.5, -6.4)* | 20.5%  (13.6, 27.0) | 20.0%  (7.0, 38.1)* | 3009.5  (2164.9, 3849.5) | -24.9%  (-35.3, -11.0)* | 12.7%  (9.1, 16.3) | 16.7%  (3.9, 34.2)* |
| Kuwait | 64.8  (44.8, 83.3) | -37.5%  (-43.0, -29.6)* | 20.5%  (14.1, 26.1) | 18.4%  (9.7, 32.0)* | 2495.4  (1816.9, 3145.6) | -22.6%  (-29.3, -13.8)* | 13.4%  (10.0, 16.4) | 22.0%  (14.4, 33.9)* |
| Lebanon | 118.5  (80.8, 155.6) | -8.8%  (-22.4, 10.0) | 20.0%  (13.6, 26.3) | 41.4%  (24.9, 66.1)* | 3401.0  (2427.7, 4311.9) | -9.2%  (-20.8, 7.7) | 13.9%  (10.2, 17.4) | 38.6%  (23.3, 61.0)* |
| Libya | 149.2  (98.0, 202.0) | 24.9%  (5.0, 51.8)* | 19.0%  (12.6, 25.2) | 27.8%  (16.4, 44.3)* | 4366.2  (3055.2, 5691.9) | 24.1%  (7.5, 45.5)* | 13.8%  (9.8, 17.6) | 35.1%  (22.9, 50.6)* |
| Morocco | 129.7  (80.4, 184.5) | 13.7%  (-8.2, 48.1) | 16.7%  (10.7, 23.2) | 56.3%  (36.4, 93.4)* | 3669.2  (2462.7, 5010.3) | 6.9%  (-10.8, 32.4) | 11.8%  (7.9, 15.7) | 63.4%  (41.5, 97.7)* |
| Palestine | 100.6  (63.1, 138.6) | -14.7%  (-28.1, 4.5) | 16.6%  (10.5, 23.0) | 24.7%  (13.3, 43.6)* | 2933.0  (1931.1, 3956.0) | -16.2%  (-27.7, -1.9)* | 11.3%  (7.5, 15.0) | 19.9%  (7.7, 36.1)* |
| Oman | 112.8  (74.7, 153.8) | 34.9%  (-1.6, 105.3) | 19.2%  (12.9, 25.4) | 114.7%  (69.1, 215.9)* | 3405.0  (2405.2, 4474.7) | 26.0%  (-5.7, 79.9) | 13.6%  (9.7, 17.3) | 99.0%  (56.4, 173.7)* |
| Qatar | 125.0  (87.7, 165.2) | -45.2%  (-54.9, -33.1)* | 23.7%  (16.7, 29.8) | 13.2%  (4.2, 25.8)* | 3303.4  (2456.9, 4237.0) | -40.1%  (-49.6, -29.7)* | 15.5%  (11.5, 18.8) | -2.2%  (-10.4, 7.5) |
| Saudi Arabia | 116.7  (78.4, 155.4) | 26.3%  (2.6, 60.7)* | 18.4%  (12.6, 24.0) | 65.8%  (41.9, 101.5)* | 3365.4  (2416.8, 4304.9) | 24.1%  (3.5, 53.0)* | 13.5%  (9.8, 16.9) | 84.0%  (58.4, 120.3)* |
| Sudan | 125.2  (75.6, 184.4) | 44.0%  (6.4, 129.4)* | 14.0%  (8.6, 19.9) | 135.8%  (84.8, 268.0)* | 3586.0  (2274.3, 5066.9) | 34.8%  (0.7, 110.4)* | 9.1%  (5.8, 12.7) | 140.3%  (81.5, 274.7)* |
| Syria | 111.5  (69.3, 161.7) | -14.0%  (-27.7, 4.0) | 12.6%  (7.8, 17.7) | -8.4%  (-17.7, 5.2) | 3324.3  (2186.4, 4572.1) | -13.1%  (-26.4, 4.2) | 8.3%  (5.4, 11.1) | -17.1%  (-27.3, -3.4)* |
| Tunisia | 91.8  (55.3, 134.4) | 9.2%  (-13.4, 43.4) | 15.0%  (9.4, 21.5) | 56.1%  (36.0, 94.7)* | 2437.9  (1600.1, 3399.3) | 9.8%  (-9.1, 36.0) | 10.4%  (7.0, 13.9) | 64.4%  (43.4, 97.1)* |
| Turkey | 75.5  (49.8, 100.7) | -35.0%  (-43.1, -24.6)* | 15.7%  (10.5, 21.0) | 21.6%  (9.8, 38.5)* | 2374.2  (1652.4, 3104.9) | -28.4%  (-36.9, -17.3)* | 10.3%  (7.1, 13.3) | 31.8%  (18.4, 51.1)* |
| United Arab Emirates | 156.9  (101.2, 215.3) | 22.8%  (-5.2, 62.5) | 18.3%  (12.2, 24.3) | 38.6%  (18.0, 67.2)* | 4358.6  (2961.6, 5716.6) | 19.8%  (-3.8, 52.3) | 14.2%  (9.9, 18.0) | 35.2%  (17.6, 61.2)* |
| Yemen | 108.6  (61.4, 165.5) | 41.5%  (-4.8, 149.5) | 10.7%  (6.4, 15.0) | 106.0%  (53.0, 242.4)* | 3141.4  (1848.0, 4594.4) | 37.2%  (-7.6, 137.4) | 7.0%  (4.4, 9.7) | 119.0%  (57.6, 269.5)* |
| Central Asia | 142.8  (91.2, 200.4) | 27.2%  (18.3, 40.2)* | 15.2%  (9.7, 21.1) | 33.2%  (25.3, 46.0)* | 3828.0  (2603.3, 5136.9) | 22.1%  (13.3, 34.8)* | 11.4%  (7.8, 15.3) | 50.7%  (40.7, 66.0)* |
| Armenia | 93.1  (56.0, 134.6) | 15.3%  (-0.9, 48.2) | 12.9%  (7.8, 18.7) | 53.0%  (32.7, 96.5)* | 2632.9  (1702.3, 3626.3) | 17.7%  (2.8, 48.6)* | 10.5%  (6.8, 14.3) | 72.9%  (50.1, 118.3)* |
| Azerbaijan | 141.8  (85.3, 204.2) | 53.6%  (32.1, 88.3)* | 14.3%  (8.7, 20.3) | 49.9%  (31.0, 82.9)* | 3779.7  (2413.1, 5141.5) | 39.6%  (20.8, 69.7)* | 11.0%  (7.0, 14.8) | 76.0%  (52.5, 115.4)* |
| Georgia | 141.9  (92.5, 198.1) | -2.8%  (-15.4, 10.3) | 16.1%  (10.5, 22.4) | 5.3%  (-8.0, 19.4) | 3753.5  (2584.8, 4945.4) | -4.6%  (-12.8, 3.2) | 12.7%  (8.8, 16.8) | 17.4%  (7.9, 27.0)* |
| Kazakhstan | 143.4  (95.1, 194.2) | 0.7%  (-8.1, 12.3) | 16.4%  (10.9, 22.2) | 15.5%  (6.2, 27.7)* | 3728.4  (2593.4, 4863.4) | -2.6%  (-10.4, 8.9) | 11.7%  (8.2, 15.2) | 22.6%  (12.6, 36.2)* |
| Kyrgyzstan | 94.8  (58.0, 137.6) | -2.0%  (-11.8, 12.0) | 12.1%  (7.5, 17.5) | 37.2%  (24.9, 55.0)* | 2686.3  (1703.7, 3732.0) | -4.3%  (-13.2, 8.9) | 8.8%  (5.7, 12.2) | 43.1%  (30.3, 62.8)* |
| Mongolia | 126.9  (76.7, 185.2) | -2.2%  (-14.5, 21.5) | 12.3%  (7.5, 17.8) | 38.4%  (24.7, 67.3)* | 3656.4  (2355.2, 5093.2) | 4.9%  (-8.4, 30.0) | 9.7%  (6.3, 13.4) | 58.9%  (42.2, 93.8)* |
| Tajikistan | 86.6  (48.3, 130.1) | 23.9%  (7.9, 48.3)* | 10.3%  (5.8, 15.4) | 39.4%  (23.8, 65.5)* | 2564.1  (1495.6, 3684.6) | 27.3%  (13.5, 50.3)* | 7.3%  (4.3, 10.5) | 59.6%  (41.8, 89.8)* |
| Turkmenistan | 162.6  (104.3, 228.5) | 24.7%  (10.6, 47.6)* | 17.8%  (11.4, 24.7) | 50.0%  (35.2, 77.6)* | 4652.6  (3155.9, 6261.9) | 27.4%  (13.0, 50.9)* | 13.5%  (9.1, 18.0) | 67.9%  (48.3, 100.3)* |
| Uzbekistan | 173.8  (105.9, 250.6) | 96.6%  (67.6, 149.3)* | 15.7%  (9.7, 22.6) | 59.3%  (40.8, 97.0)* | 4522.3  (2943.5, 6181.7) | 73.3%  (48.2, 117.9)* | 12.8%  (8.5, 17.5) | 90.2%  (67.2, 135.7)* |
| South Asia | 51.5  (29.9, 77.0) | 158.3%  (77.3, 403.9)* | 5.1%  (3.0, 7.6) | 277.0%  (157.1, 637.8)* | 1609.0  (976.6, 2298.8) | 165.9%  (83.3, 400.7)* | 4.1%  (2.5, 5.8) | 328.9%  (196.0, 715.0)* |
| Bangladesh | 31.0  (13.6, 54.3) | 190.3%  (73.7, 774.0)* | 3.9%  (1.8, 6.8) | 445.2%  (232.8, 1542.7)* | 1089.6  (543.1, 1775.3) | 231.8%  (97.2, 915.8)* | 3.3%  (1.7, 5.3) | 578.8%  (304.0, 1973.3)* |
| Bhutan | 46.8  (23.2, 76.3) | 57.5%  (4.6, 238.4)* | 6.6%  (3.4, 10.5) | 202.0%  (112.3, 530.0)* | 1480.7  (792.0, 2291.1) | 62.1%  (10.5, 249.7)* | 5.1%  (2.8, 7.7) | 229.3%  (128.0, 602.5)* |
| India | 49.9  (29.4, 73.5) | 166.1%  (81.8, 404.4)* | 4.9%  (2.9, 7.2) | 292.6%  (168.2, 639.5)* | 1559.6  (957.5, 2210.2) | 167.8%  (83.5, 393.6)* | 3.9%  (2.5, 5.6) | 330.3%  (197.2, 691.4)* |
| Nepal | 42.5  (19.5, 71.1) | 172.2%  (78.9, 511.5)* | 4.7%  (2.2, 7.8) | 356.1%  (204.6, 904.9)* | 1368.5  (705.6, 2162.6) | 179.8%  (81.6, 517.0)* | 3.9%  (2.0, 6.1) | 457.4%  (265.6, 1119.5)* |
| Pakistan | 94.8  (51.9, 148.4) | 168.0%  (76.8, 514.7)* | 8.1%  (4.6, 12.2) | 200.5%  (108.8, 581.5)* | 2694.2  (1562.8, 4012.9) | 158.9%  (72.3, 487.8)* | 5.9%  (3.5, 8.6) | 251.1%  (140.1, 693.8)* |
| Southeast Asia | 56.7  (33.6, 82.7) | 89.3%  (40.9, 216.5)* | 7.1%  (4.2, 10.4) | 170.9%  (102.6, 348.1)* | 1971.7  (1252.2, 2730.1) | 97.1%  (47.6, 217.3)* | 6.4%  (4.1, 8.8) | 202.6%  (128.0, 388.4)* |
| Cambodia | 33.1  (14.7, 56.9) | 31.4%  (-6.4, 163.2) | 3.4%  (1.5, 5.9) | 123.3%  (62.2, 347.3)* | 1123.5  (524.3, 1862.8) | 38.5%  (-0.8, 175.6) | 3.1%  (1.4, 5.0) | 157.9%  (88.1, 409.7)* |
| Indonesia | 61.3  (35.1, 93.1) | 102.9%  (49.6, 261.8)* | 6.8%  (3.9, 10.2) | 170.4%  (100.2, 377.0)* | 2220.2  (1399.8, 3127.0) | 114.3%  (58.3, 276.7)* | 6.8%  (4.3, 9.5) | 231.5%  (145.2, 480.7)* |
| Laos | 65.4  (34.8, 102.0) | 46.5%  (-3.3, 215.7) | 6.3%  (3.4, 9.6) | 171.7%  (83.9, 480.1)* | 2228.7  (1229.5, 3285.4) | 60.5%  (4.3, 251.3)* | 5.3%  (3.1, 7.8) | 223.8%  (114.4, 594.2)* |
| Malaysia | 72.7  (48.1, 98.6) | 23.0%  (-2.0, 77.2) | 9.9%  (6.6, 13.3) | 57.5%  (27.0, 125.3)* | 2457.9  (1762.4, 3200.8) | 26.7%  (4.2, 74.0)* | 9.6%  (7.0, 12.3) | 58.7%  (32.0, 117.5)* |
| Maldives | 34.1  (19.2, 52.7) | -11.2%  (-37.6, 84.4) | 8.0%  (4.6, 12.3) | 146.6%  (74.0, 414.0)* | 1344.7  (842.0, 1924.7) | 8.8%  (-25.3, 128.5) | 7.3%  (4.6, 10.2) | 166.7%  (84.2, 473.0)* |
| Mauritius | 117.5  (76.8, 160.1) | 20.0%  (1.2, 57.6)* | 16.8%  (11.1, 22.5) | 67.9%  (43.2, 119.7)* | 3770.0  (2641.1, 4928.5) | 25.1%  (7.2, 61.5)* | 13.6%  (9.6, 17.5) | 58.5%  (36.6, 104.7)* |
| Myanmar | 45.0  (22.1, 74.2) | 50.3%  (-3.1, 251.3) | 4.5%  (2.3, 7.3) | 164.5%  (77.3, 499.7)* | 1546.1  (833.2, 2393.6) | 61.9%  (5.1, 255.6)* | 3.9%  (2.2, 6.0) | 206.0%  (102.8, 569.9)* |
| Philippines | 94.5  (55.1, 139.3) | 157.5%  (85.1, 324.8)* | 9.2%  (5.3, 13.1) | 166.7%  (101.3, 331.5)* | 3027.7  (1821.9, 4230.1) | 147.6%  (83.0, 289.3)* | 8.3%  (5.1, 11.4) | 193.1%  (123.2, 357.0)* |
| Sri Lanka | 51.3  (27.7, 77.7) | 45.5%  (3.4, 132.7)* | 8.7%  (4.8, 12.7) | 141.5%  (83.6, 275.5)* | 1586.1  (932.1, 2299.9) | 44.8%  (8.4, 113.7)* | 7.0%  (4.2, 9.7) | 137.6%  (85.8, 246.3)* |
| Seychelles | 75.3  (47.0, 107.6) | -2.5%  (-14.3, 19.4) | 9.7%  (6.1, 13.9) | 17.7%  (5.1, 43.8)* | 2659.6  (1850.7, 3577.5) | 5.2%  (-6.5, 26.8) | 9.5%  (6.7, 12.6) | 24.9%  (11.9, 49.1)* |
| Thailand | 43.8  (28.0, 61.7) | 66.3%  (13.8, 241.8)* | 8.4%  (5.5, 11.7) | 177.2%  (92.4, 467.7)* | 1634.6  (1126.1, 2186.1) | 85.2%  (30.5, 258.9)* | 6.9%  (4.9, 9.0) | 172.3%  (93.8, 429.2)* |
| Timor-Leste | 26.6  (8.7, 53.3) | 11.8%  (-18.3, 95.5) | 3.0%  (1.0, 5.8) | 71.2%  (33.1, 192.0)* | 925.2  (344.2, 1702.1) | 19.6%  (-11.8, 106.4) | 2.7%  (1.0, 5.0) | 119.9%  (65.5, 266.9)* |
| Vietnam | 32.4  (15.2, 55.5) | 83.4%  (30.7, 268.3)* | 4.5%  (2.1, 7.7) | 148.7%  (81.6, 406.4)* | 1022.3  (483.5, 1697.4) | 87.4%  (33.5, 265.7)* | 3.9%  (1.9, 6.3) | 173.7%  (100.0, 431.0)* |
| East Asia | 43.9  (21.5, 72.5) | 49.8%  (13.7, 175.2)* | 7.0%  (3.4, 11.6) | 146.9%  (88.4, 352.4)* | 1307.7  (704.7, 2028.5) | 59.1%  (17.3, 201.2)* | 5.9%  (3.2, 9.0) | 176.1%  (105.9, 421.2)* |
| China | 44.2  (21.7, 72.9) | 53.4%  (15.1, 193.3)* | 7.1%  (3.4, 11.7) | 155.2%  (92.6, 385.2)* | 1312.5  (704.8, 2034.2) | 62.8%  (18.4, 222.7)* | 5.9%  (3.2, 9.1) | 186.4%  (110.7, 466.7)* |
| North Korea | 26.1  (6.0, 61.1) | -2.9%  (-30.7, 19.0) | 3.0%  (0.7, 6.8) | -3.0%  (-28.3, 10.4) | 768.1  (179.9, 1747.6) | -3.3%  (-32.9, 16.2) | 2.4%  (0.6, 5.4) | -5.1%  (-31.7, 8.0) |
| Taiwan  (Province of China) | 42.4  (26.3, 60.1) | -25.6%  (-33.9, -10.8)* | 8.8%  (5.5, 12.5) | 13.4%  (2.1, 36.8)* | 1531.0  (1046.6, 2054.7) | -9.6%  (-21.2, 13.2) | 7.8%  (5.5, 10.1) | 19.1%  (4.9, 48.4)* |
| Oceania | 171.7  (104.0, 251.2) | 34.2%  (12.9, 80.1)* | 11.2%  (6.9, 16.3) | 50.8%  (29.5, 99.1)* | 5779.9  (3671.5, 8102.7) | 35.5%  (14.2, 77.7)* | 10.0%  (6.5, 13.9) | 54.9%  (33.1, 101.7)* |
| American Samoa | 181.4  (129.0, 235.5) | 22.4%  (6.9, 46.1)* | 20.2%  (14.6, 25.4) | 30.1%  (18.6, 50.0)* | 6301.3  (4892.7, 7654.4) | 20.5%  (9.1, 35.7)* | 19.6%  (15.5, 23.3) | 26.7%  (17.1, 39.9)* |
| Federated States of Micronesia | 193.4  (122.9, 267.4) | 23.7%  (0.5, 57.9)* | 16.3%  (10.4, 22.3) | 45.5%  (24.5, 75.1)* | 6803.5  (4695.4, 8829.0) | 22.8%  (0.2, 52.9)* | 16.3%  (11.3, 21.1) | 48.3%  (26.0, 75.9)* |
| Fiji | 294.4  (209.5, 379.8) | 69.4%  (33.9, 138.1)* | 26.1%  (18.9, 32.6) | 71.1%  (40.8, 131.2)* | 8895.7  (6817.2, 11004.9) | 61.9%  (29.7, 118.0)* | 22.4%  (17.2, 27.0) | 68.6%  (37.5, 123.6)* |
| Guam | 149.0  (103.4, 191.6) | 25.5%  (10.7, 46.7)* | 19.9%  (14.1, 25.3) | 27.0%  (14.1, 47.0)* | 5052.1  (3729.8, 6294.9) | 35.4%  (22.3, 53.5)* | 16.8%  (12.6, 20.4) | 26.7%  (15.9, 43.1)* |
| Kiribati | 241.9  (157.1, 335.3) | 42.5%  (17.1, 87.3)* | 16.5%  (10.9, 22.5) | 63.2%  (42.5, 106.8)* | 8842.8  (6352.3, 11436.0) | 42.7%  (19.1, 82.1)* | 16.5%  (12.0, 21.0) | 73.9%  (51.0, 115.9)* |
| Marshall Islands | 195.0  (114.6, 286.2) | 61.8%  (22.3, 167.6)* | 14.3%  (8.4, 20.6) | 80.4%  (42.2, 196.6)* | 6867.6  (4496.9, 9630.3) | 74.1%  (31.9, 184.9)* | 14.5%  (9.6, 19.6) | 91.5%  (50.2, 219.9)* |
| Northern Mariana Islands | 103.7  (69.4, 139.5) | -0.2%  (-15.9, 21.1) | 16.3%  (11.0, 21.6) | 20.9%  (11.0, 36.1)* | 3628.7  (2618.9, 4675.1) | 6.1%  (-7.8, 22.8) | 14.7%  (10.8, 18.3) | 16.3%  (8.1, 29.3)* |
| Papua New Guinea | 143.9  (73.5, 224.8) | 26.1%  (3.2, 82.5)* | 8.3%  (4.4, 13.0) | 47.4%  (25.5, 109.5)* | 5183.5  (2890.6, 7808.2) | 30.4%  (5.7, 88.6)* | 8.1%  (4.7, 11.9) | 57.4%  (31.7, 124.9)* |
| Samoa | 147.8  (91.6, 203.3) | 20.0%  (-0.9, 47.9) | 17.2%  (10.8, 23.2) | 26.7%  (15.7, 44.8)* | 4730.2  (3295.3, 6123.6) | 10.7%  (-5.3, 30.7) | 15.7%  (11.2, 19.7) | 26.2%  (15.2, 42.3)* |
| Solomon Islands | 140.8  (77.0, 207.6) | 30.0%  (4.1, 89.9)* | 11.2%  (6.3, 16.7) | 55.6%  (31.6, 120.2)* | 5124.1  (3188.8, 7100.3) | 35.2%  (7.7, 93.1)* | 11.4%  (7.4, 15.8) | 66.4%  (38.9, 133.2)* |
| Tonga | 144.9  (92.5, 201.3) | 33.0%  (11.7, 65.8)* | 15.9%  (10.4, 21.5) | 49.7%  (32.4, 79.2)* | 5015.2  (3617.2, 6454.3) | 31.2%  (14.7, 53.1)* | 15.4%  (11.3, 19.1) | 43.1%  (30.3, 64.4)* |
| Vanuatu | 162.4  (90.6, 249.5) | 24.3%  (-3.4, 74.9) | 12.0%  (7.0, 17.7) | 37.1%  (19.4, 77.2)* | 5620.6  (3443.2, 8217.4) | 29.3%  (0.5, 80.9)* | 11.8%  (7.7, 16.2) | 44.6%  (24.4, 86.6)* |
| High-income Asia Pacific | 15.4  (6.2, 27.0) | -39.2%  (-46.3, -22.9)* | 4.6%  (1.9, 8.0) | 5.1%  (-6.6, 33.7) | 576.0  (253.9, 973.1) | -23.9%  (-32.7, -3.1)* | 3.5%  (1.6, 5.8) | 3.6%  (-7.2, 30.2) |
| Brunei | 64.6  (35.1, 96.5) | 2.9%  (-20.4, 69.0) | 9.4%  (5.2, 14.2) | 54.9%  (22.8, 152.8)* | 2029.9  (1174.0, 2973.3) | 10.6%  (-13.8, 81.1) | 8.0%  (4.7, 11.5) | 44.6%  (15.0, 138.1)* |
| Japan | 14.4  (5.9, 25.6) | -33.0%  (-40.9, -14.4)* | 4.4%  (1.8, 7.8) | 7.3%  (-5.5, 37.3) | 538.2  (235.9, 915.6) | -16.0%  (-24.8, 4.8) | 3.3%  (1.5, 5.5) | 4.0%  (-6.3, 28.9) |
| Singapore | 22.6  (12.0, 35.3) | -30.0%  (-46.4, 21.1) | 7.5%  (4.0, 11.8) | 57.5%  (21.4, 173.2)* | 860.4  (500.9, 1269.0) | -11.1%  (-32.8, 57.5) | 5.8%  (3.5, 8.3) | 42.3%  (8.7, 153.6)* |
| South Korea | 19.7  (7.8, 35.0) | -58.1%  (-64.7, -42.2)* | 5.1%  (2.0, 9.0) | -3.7%  (-17.3, 31.7) | 665.6  (283.3, 1141.0) | -48.8%  (-56.5, -30.8)* | 3.9%  (1.7, 6.5) | -8.2%  (-19.2, 20.2) |
| High-income North America | 69.1  (47.1, 89.5) | -8.6%  (-16.9, 5.0) | 13.5%  (9.2, 17.5) | 16.4%  (6.1, 33.7)* | 2262.5  (1661.3, 2847.2) | 2.7%  (-6.7, 17.4) | 9.5%  (7.0, 11.7) | 17.7%  (7.5, 33.9)* |
| Canada | 45.2  (29.7, 60.9) | -21.2%  (-31.9, -1.9)* | 11.3%  (7.5, 15.1) | 16.9%  (2.4, 44.5)* | 1484.6  (1037.6, 1959.8) | -13.0%  (-22.9, 4.1) | 7.7%  (5.5, 9.8) | 7.2%  (-4.0, 27.9) |
| Greenland | 60.3  (34.9, 88.5) | -26.6%  (-35.6, -12.8)* | 7.8%  (4.5, 11.5) | 18.7%  (5.3, 41.6)* | 1757.1  (1088.6, 2493.9) | -27.4%  (-35.2, -14.8)* | 5.9%  (3.6, 8.2) | 15.5%  (3.8, 35.2)* |
| United States | 72.0  (49.5, 92.9) | -7.1%  (-15.3, 7.0) | 13.8%  (9.4, 17.8) | 16.4%  (6.4, 33.6)* | 2355.1  (1732.3, 2959.2) | 4.4%  (-5.1, 19.1) | 9.7%  (7.2, 11.9) | 18.7%  (8.6, 34.6)* |
| Western Europe | 38.8  (23.6, 56.1) | -33.8%  (-39.1, -23.8)* | 9.4%  (5.7, 13.6) | 6.9%  (-1.2, 22.5) | 1200.8  (773.3, 1676.3) | -21.5%  (-29.3, -8.1)* | 6.5%  (4.3, 8.8) | 5.1%  (-3.3, 21.7) |
| Andorra | 34.6  (21.3, 49.7) | -10.6%  (-28.3, 18.7) | 9.1%  (5.7, 12.9) | 25.3%  (7.9, 62.4)* | 1221.7  (810.8, 1686.2) | 5.4%  (-13.0, 39.5) | 6.8%  (4.7, 9.1) | 27.9%  (10.2, 63.7)* |
| Austria | 45.2  (27.2, 65.3) | -24.7%  (-34.3, -5.9)* | 10.8%  (6.6, 15.5) | 25.5%  (11.0, 55.9)* | 1249.9  (809.1, 1761.5) | -17.1%  (-26.9, 1.2) | 6.7%  (4.4, 9.2) | 12.9%  (1.4, 36.1)* |
| Belgium | 34.5  (20.9, 49.3) | -35.1%  (-43.2, -21.8)* | 8.0%  (4.8, 11.4) | 2.3%  (-9.3, 22.8) | 1182.8  (764.7, 1663.7) | -19.1%  (-28.7, -2.5)* | 6.1%  (4.0, 8.2) | 5.6%  (-5.3, 26.1) |
| Cyprus | 43.6  (25.8, 63.9) | -17.0%  (-32.1, 18.3) | 10.5%  (6.3, 15.4) | 38.1%  (15.8, 96.3)* | 1368.5  (883.4, 1945.2) | -0.6%  (-17.8, 41.3) | 7.5%  (4.9, 10.3) | 37.0%  (16.0, 92.9)* |
| Denmark | 34.4  (20.3, 50.5) | -38.5%  (-46.3, -25.0)* | 7.4%  (4.5, 10.9) | -2.8%  (-14.0, 17.2) | 1254.2  (792.2, 1782.3) | -19.9%  (-30.7, -1.0)* | 6.5%  (4.3, 8.8) | 8.7%  (-3.7, 32.8) |
| Finland | 45.3  (26.9, 66.4) | -33.8%  (-42.6, -18.6)* | 10.5%  (6.3, 15.3) | 11.3%  (-3.0, 36.2) | 1410.2  (924.0, 1979.7) | -24.3%  (-34.4, -6.5)* | 7.2%  (4.8, 9.8) | 4.4%  (-6.9, 26.7) |
| France | 28.0  (16.9, 40.9) | -29.2%  (-37.7, -13.9)* | 7.5%  (4.5, 11.0) | 18.0%  (4.9, 41.5)* | 918.8  (591.4, 1298.7) | -13.2%  (-23.1, 3.7) | 5.2%  (3.4, 7.0) | 16.4%  (5.5, 37.7)* |
| Germany | 48.7  (28.5, 72.8) | -32.1%  (-40.7, -18.7)* | 10.6%  (6.3, 15.2) | 4.7%  (-5.6, 23.9) | 1356.7  (873.4, 1904.6) | -23.5%  (-32.8, -7.8)* | 6.9%  (4.5, 9.5) | 0.6%  (-8.8, 19.6) |
| Greece | 48.6  (29.0, 70.2) | -13.5%  (-23.0, 5.4) | 10.8%  (6.4, 15.5) | 15.9%  (4.6, 41.4)* | 1502.8  (962.7, 2093.2) | -0.7%  (-11.0, 19.7) | 7.8%  (5.0, 10.6) | 19.0%  (7.5, 42.8)* |
| Iceland | 38.9  (23.7, 55.8) | -31.5%  (-38.7, -18.1)* | 10.2%  (6.3, 14.8) | 5.4%  (-5.3, 24.2) | 1340.4  (889.4, 1867.2) | -15.8%  (-26.2, 1.5) | 7.6%  (5.2, 10.0) | 9.3%  (-1.8, 30.8) |
| Ireland | 37.5  (21.8, 55.2) | -45.9%  (-52.1, -34.0)* | 9.0%  (5.3, 13.0) | 1.0%  (-9.6, 23.2) | 1280.2  (811.6, 1808.8) | -30.7%  (-40.1, -14.6)* | 6.9%  (4.5, 9.3) | -1.8%  (-12.4, 19.4) |
| Israel | 37.7  (22.2, 54.5) | -35.2%  (-44.2, -17.6)* | 10.0%  (5.9, 14.5) | 6.6%  (-7.5, 34.9) | 1173.7  (726.8, 1673.7) | -21.4%  (-31.4, -1.8)* | 6.7%  (4.3, 9.2) | 5.9%  (-5.8, 31.9) |
| Italy | 36.3  (21.6, 53.2) | -33.1%  (-41.2, -18.3)* | 9.9%  (5.9, 14.3) | 13.9%  (1.0, 38.6)* | 1055.4  (670.6, 1498.4) | -24.6%  (-31.6, -13.1)* | 6.2%  (4.0, 8.5) | 5.8%  (-2.2, 21.3) |
| Luxembourg | 41.8  (26.7, 59.3) | -34.4%  (-44.3, -18.8)* | 9.7%  (6.2, 13.6) | 9.5%  (-4.8, 33.9) | 1413.3  (966.3, 1913.7) | -20.2%  (-31.0, -3.9)* | 7.4%  (5.3, 9.6) | 11.7%  (-0.7, 33.0) |
| Malta | 50.9  (30.8, 73.5) | -24.1%  (-33.8, -4.1)* | 11.3%  (6.8, 16.1) | 13.7%  (-0.3, 43.2) | 1688.6  (1094.3, 2345.4) | -6.0%  (-19.3, 18.5) | 8.8%  (5.9, 11.7) | 17.0%  (2.4, 47.0)* |
| Netherlands | 33.4  (19.6, 49.5) | -36.2%  (-42.9, -24.1)* | 7.7%  (4.4, 11.3) | -6.8%  (-15.5, 9.7) | 1169.7  (742.3, 1683.2) | -18.4%  (-28.6, 0.2) | 6.3%  (4.1, 8.6) | 3.9%  (-6.8, 27.0) |
| Norway | 31.8  (19.2, 46.7) | -39.5%  (-47.3, -26.1)* | 8.0%  (4.8, 11.6) | -1.5%  (-14.0, 19.5) | 1104.9  (709.5, 1564.2) | -25.9%  (-36.1, -8.7)* | 5.9%  (4.0, 8.1) | -0.5%  (-13.5, 22.0) |
| Portugal | 40.8  (24.2, 59.3) | -34.0%  (-43.8, -13.1)* | 9.4%  (5.6, 13.5) | 23.4%  (6.2, 61.5)* | 1433.0  (905.0, 2037.9) | -16.8%  (-29.1, 6.4) | 7.6%  (5.0, 10.3) | 27.1%  (11.0, 63.0)* |
| Spain | 36.4  (22.1, 52.7) | -35.9%  (-42.5, -25.1)* | 9.8%  (6.0, 14.1) | 8.1%  (-2.6, 25.8) | 1133.7  (728.7, 1564.4) | -23.6%  (-31.3, -10.9)* | 6.7%  (4.4, 9.0) | 7.1%  (-1.5, 23.8) |
| Sweden | 36.6  (21.6, 53.6) | -28.3%  (-36.6, -12.0)* | 9.2%  (5.4, 13.6) | 8.6%  (-3.4, 33.7) | 1169.1  (735.6, 1658.6) | -14.1%  (-24.9, 6.0) | 6.5%  (4.2, 8.9) | 9.3%  (-2.3, 34.1) |
| Switzerland | 28.5  (16.5, 42.7) | -38.9%  (-46.8, -27.4)* | 8.4%  (4.8, 12.4) | 4.8%  (-7.8, 23.4) | 950.0  (591.2, 1366.3) | -25.4%  (-33.6, -12.9)* | 5.6%  (3.6, 7.7) | 3.1%  (-5.3, 19.4) |
| United Kingdom | 41.8  (25.6, 59.6) | -38.6%  (-44.4, -28.9)* | 9.3%  (5.7, 13.3) | -4.9%  (-13.8, 10.4) | 1377.1  (893.9, 1904.1) | -23.8%  (-32.3, -9.7)* | 6.9%  (4.6, 9.2) | -3.0%  (-12.0, 14.1) |
| Australasia | 44.2  (28.6, 60.7) | -28.4%  (-39.1, -9.3)* | 11.2%  (7.4, 15.2) | 15.5%  (1.0, 44.8)* | 1393.7  (961.0, 1855.8) | -17.2%  (-28.7, 2.5) | 7.2%  (5.0, 9.3) | 8.3%  (-4.6, 33.7) |
| Australia | 44.1  (28.7, 60.3) | -27.8%  (-39.3, -8.0)* | 11.3%  (7.4, 15.2) | 15.7%  (0.4, 46.2)* | 1389.1  (957.0, 1844.8) | -16.2%  (-28.6, 4.7) | 7.3%  (5.1, 9.3) | 8.8%  (-5.3, 35.4) |
| New Zealand | 44.7  (28.3, 62.4) | -31.5%  (-41.0, -14.6)* | 10.7%  (6.8, 14.9) | 13.0%  (-1.7, 38.7) | 1416.4  (948.3, 1913.7) | -22.0%  (-31.8, -4.6)* | 6.9%  (4.7, 9.2) | 5.1%  (-7.3, 26.7) |

DALY= disability-adjusted life year; PAF= population attributable fraction; SDI= Socio-demographic Index; *Changes that are statistically significant.
